# A new synthetic toll-like receptor 1/2 ligand is an efficient adjuvant for peptide vaccination in a human volunteer

**DOI:** 10.1186/s40425-019-0796-5

**Published:** 2019-11-15

**Authors:** Hans-Georg Rammensee, Karl-Heinz Wiesmüller, P. Anoop Chandran, Henning Zelba, Elisa Rusch, Cécile Gouttefangeas, Daniel J. Kowalewski, Moreno Di Marco, Sebastian P. Haen, Juliane S. Walz, Yamel Cardona Gloria, Johanna Bödder, Jill-Marie Schertel, Antje Tunger, Luise Müller, Maximilian Kießler, Rebekka Wehner, Marc Schmitz, Meike Jakobi, Nicole Schneiderhan-Marra, Reinhild Klein, Karoline Laske, Kerstin Artzner, Linus Backert, Heiko Schuster, Johannes Schwenck, Alexander N. R. Weber, Bernd J. Pichler, Manfred Kneilling, Christian la Fougère, Stephan Forchhammer, Gisela Metzler, Jürgen Bauer, Benjamin Weide, Wilfried Schippert, Stefan Stevanović, Markus W. Löffler

**Affiliations:** 1 Department of Immunology, Institute for Cell Biology, University of Tübingen, Auf der Morgenstelle 15, 72076 Tübingen, Germany; 2 German Cancer Consortium (DKTK) and German Cancer Research Center (DKFZ) partner site Tübingen, Tübingen, Germany; 3 Cluster of Excellence iFIT (EXC2180) “Image-Guided and Functionally Instructed Tumor Therapies”, University of Tübingen, Tubingen, Germany; 4 EMC microcollections GmbH, Tübingen, Germany; 5 Present address: Immatics Biotechnologies GmbH, Tübingen, Germany; 6 Department of Oncology, Hematology, Immunology, Rheumatology and Pulmonology, University Hospital of Tübingen, Tübingen, Germany; 7 Faculty of Medicine Carl Gustav Carus, Institute of Immunology, Technische Universität Dresden, Dresden, Germany; 8 National Center for Tumor Diseases (NCT), Partner Site Dresden, Germany: German Cancer Research Center (DKFZ), Heidelberg, Germany, Faculty of Medicine and University Hospital Carl Gustav Carus, Technische Universität Dresden, Dresden, Germany and Helmholtz Association/ Helmholtz-Zentrum Dresden-Rossendorf (HZDR), Dresden, Germany; 9 German Cancer Consortium (DKTK), Partner Site Dresden, and German Cancer Research Center (DKFZ), Heidelberg, Germany; 10 NMI Natural and Medical Sciences Institute at the University of Tübingen, Reutlingen, Germany; 11 Department of Nuclear Medicine and Clinical Molecular Imaging, University Hospital of Tübingen, Tübingen, Germany; 12 Werner Siemens Imaging Center, Medical Faculty, University of Tübingen, Tübingen, Germany; 13 Department of Dermatology, University Hospital of Tübingen, Tübingen, Germany; 14 Department of General, Visceral and Transplant Surgery, University Hospital of Tübingen, Tübingen, Germany; 15 Department of Clinical Pharmacology, University Hospital Tübingen, Tübingen, Germany

**Keywords:** Adjuvant, Lipopeptide, TLR1/2 ligand, Immunotherapy, Vaccines

## Abstract

**Background:**

We previously showed that the bacterial lipopeptide Pam_3_Cys-Ser-Ser, meanwhile established as a toll-like receptor (TLR) 1/2 ligand, acts as a strong adjuvant for the induction of virus specific CD8^+^ T cells in mice, when covalently coupled to a synthetic peptide.

**Case presentation:**

We now designed a new water-soluble synthetic Pam_3_Cys-derivative, named XS15 and characterized it in vitro by a TLR2 NF-κB luciferase reporter assay. Further, the capacity of XS15 to activate immune cells and stimulate peptide-specific CD8^+^ T and NK cells by 6-sulfo LacNAc^+^ monocytes was assessed by flow cytometry as well as cytokine induction using immunoassays. The induction of a functional immune response after vaccination of a volunteer with viral peptides was assessed by ELISpot assay and flow cytometry in peripheral blood cells and infiltrating cells at the vaccination site, as well as by immunohistochemistry and imaging.

XS15 induced strong ex vivo CD8^+^ and T_H_1 CD4^+^ responses in a human volunteer upon a single injection of XS15 mixed to uncoupled peptides in a water-in-oil emulsion (Montanide™ ISA51 VG). A granuloma formed locally at the injection site containing highly activated functional CD4^+^ and CD8^+^ effector memory T cells. The total number of vaccine peptide-specific functional T cells was experimentally assessed and estimated to be 3.0 × 10^5^ in the granuloma and 20.5 × 10^6^ in peripheral blood.

**Conclusion:**

Thus, in one volunteer we show a granuloma forming by peptides combined with an efficient adjuvant in a water-in-oil-emulsion, inducing antigen specific T cells detectable in circulation and at the vaccination site, after one single vaccination only. The ex vivo T cell responses in peripheral blood were detectable for more than one year and could be strongly boosted by a second vaccination. Hence, XS15 is a promising adjuvant candidate for peptide vaccination, in particular for tumor peptide vaccines in a personalized setting.

## Background

Cancer immunotherapy gained significant impact by introduction of immune checkpoint inhibition (ICI) into clinical practice, whereby immune responses against tumor antigens are stimulated, predominantly against neoantigens [1, 2]. However, severe immune related adverse events are also observed, possibly due to immune responses against normal self-antigens. In many cancers, particularly those with low mutational burden, ICI is often unsuccessful [3]. A robust vaccination approach would therefore be highly desirable, enabling the de novo induction of immune responses against specific tumor antigens. Hitherto, all tumor vaccination trials have either failed in phase III [4] or showed only marginal benefits. Particularly peptide-based cancer vaccines have shown limited success, although objective clinical responses correlating with immune responses were repeatedly reported (e.g. [5]). One underlying reason is the lack of efficient adjuvants. Among the most effective peptide vaccination methods tested in humans is subcutaneous injection of peptides emulsified in Montanide, a water-in-oil-emulsion, combined with the TLR9 ligand CpG [6]. Unfortunately, CpG manufactured according to good manufacturing practice (GMP) is not commercially available. In addition, Montanide application frequently causes a long-lasting granuloma at the injection site. Such granulomas have been found to induce CD8^+^ T cell sequestration, dysfunction and deletion in mice [7]. Although many additional promising candidates have entered preclinical or clinical development [8–10], currently, only very few adjuvants are available for human use, most with insufficient efficiency.

The TLR1/2 ligand Pam_3_Cys-Ser-Ser is very efficient for inducing CD8^+^ T cells in mice, when covalently coupled to synthetic peptides [11] and injected intraperitoneally. Pam_3_Cys-based vaccines have been clinically used, in particular for Borreliosis (reviewed in [9]). Most Pam_3_Cys-conjugates, however, are not water-soluble, difficult to purify by high performance liquid chromatography, extremely challenging for GMP production, and therefore inappropriate for (personalized) clinical vaccination approaches. We hence sought to design a Pam_3_Cys derived TLR1/2 ligand that 1) is water-soluble and 2) GMP-amenable, 3) non-toxic and 4) effective for inducing T cells specific for peptides only admixed (i.e. not covalently coupled to the TLR1/2-ligand, when applied in vivo). We here present XS15 as a promising adjuvant candidate fulfilling all these basic requirements.

## Materials and methods

Sections with additional details available in *Supplementary Materials and Method*s (Additional file 1) are marked by an asterisk in the respective headings.

### Synthesis of Pam_3_Cys-GDPKHPKSF (XS15)

The peptide GDPKHPKSF was synthesized by fully automated solid phase synthesis and Fmoc/tBu chemistry on chlorotrityl-resin. To generate the triple-chain lipopeptide, the peptide resin was elongated with the unusual amino acid Fmoc-S-(2,3-dihydroxy-2(RS)-propyl)-cysteine followed by esterification on solid phase with palmitic acid. After Fmoc-deprotection, the lipopeptide was modified by N-palmitoylation.

### Dual-luciferase assay^*^

HEK293T cells were co-transfected with a human TLR2 plasmid and firefly luciferase under a synthetic NF-κB promoter and the constitutive Renilla luciferase reporter. Cells were stimulated with Pam_3_CysSK_4_ and XS15. Lysates were analysed using the Dual-Luciferase reporter assay kit (Promega, Madison, MI).

### HEK-dual hTLR2 analysis with TLR1, TLR2 and TLR6 blocking antibodies^*^

HEK-Dual hTLR2 cells (InvivoGen, San Diego, CA) were incubated with TLR1, TLR2 and TLR6 blocking antibodies (InvivoGen) or isotype control and stimuli added. SEAP levels (driven by an NF-κB promoter) were measured in supernatants (QUANTI-Blue; InvivoGen) [12].

### Case presentation

The healthy volunteer described herein is a white male of European descent, aged 62 years at first vaccination. The individual remained healthy during the described period and reported no significant prior medical history or ongoing disease, except for preexisting arterial hypertension treated by irbesartan (150 mg) and lercanidipine hydrochloride (5 mg) as well as acetyl salicylic acid (100 mg) taken for prophylactic purposes (all medication taken once daily).

### Ethical considerations

The described vaccinated individual performed all vaccinations as self-experimentation. This was undertaken voluntarily by an investigator and designer of the research himself on his own person. Interventions by physicians involved were exclusively performed after obtaining informed consent and assuring a reasonable risk-benefit assessment. In mouse toxicology studies the no-observable-effect level (NOEL) [15] was tested by administering up to 50 μg XS15, without observing any toxicity.

Since any coercion or dependency can be excluded in this case, no other party is to be protected from unethical behavior [13]. Respective conduct is widely considered appropriate and as an ethically and legally legitimate form of experimentation [13]. Self-experimentation is historically established and common among scientists, offering a route to valuable human experimentation, when performed properly [13, 14].

### Human samples

Anticoagulated whole blood (heparin/ citrate) or buffy coats (Center for Clinical Transfusion Medicine GmbH, Tübingen) were obtained from healthy donors after informed consent and from one vaccinated volunteer.

### Isolation of PBMCs

Peripheral blood mononuclear cells (PBMCs) were isolated by density centrifugation and either used fresh or after liquid nitrogen storage [16].

### Synthetic peptides, monomers and multimers^*^

Automated peptide synthesis was performed in-house (ABI 433A; Applied Biosystems, Foster City, CA). Lyophilized peptides (see Tables 1 and 2) were diluted in DMSO or water/ DMSO for T-cell assays and monomer refolding, respectively. The former was performed by conventional refolding as described before [17, 18], whereas ADV-Hex HLA-A*01-peptide and FLU-NCAP HLA-B*08-peptide monomers were generated by exchange of an HLA-B*08 UV labile monomer [19]. Multimers were generated by incubating monomers with streptavidin-PE/ streptavidin-APC (Biolegend, San Diego, CA) together with glycerol and human serum albumin [20].

**Table 1:** Synthetic Peptides, first vaccination and immunomonitoring

ID	Amino acid (AA) sequence	HLA-restriction	Source protein	AA position within source protein
ADV-Hex	LTDLGQNLLY	HLA-A*01	Human adenovirus 5, Hexon protein (HAdV-5, hex)	885–894
FLU-NCAP	ELRSRYWAI	HLA-B*08	Influenza A virus, Nucleoprotein (H1N1, np)	380–388
EBV-GP350	PRPVSRFLGNNSILY	HLA-DR	Epstein-Barr virus, Envelope Glycoprotein GP350 (EBV, gp350)	268–282
	Control peptides used for immunomonitoring only			
HIV-A*01	GSEELRSLY	HLA-A*01	Human Immunodeficiency virus 1, Gag-Pol polyprotein (HIV-1, gag-pol)	71–79
HIV-B*08	GGKKKYKL	HLA-B*08	Human Immunodeficiency virus 1, Gag-Pol polyprotein (HIV-1, gag-pol)	24–31
Fil-A	ETVITVDTKAAGKGK	HLA-DR	Human Filamin A protein (FLNA)	1669–1683

**Table 2:** Synthetic Peptides, second vaccination and immunomonitoring

ID	Amino acid sequence	HLA-restriction	Source protein, HLA-restriction	AA position within source protein
CMV-VPAP	VTEHDTLLY	HLA-A*01	Human cytomegalovirus, DNA polymerase processivity factor (hCMV, VPAP)	245–253
CMV-pp65	YSEHPTFTSQY	HLA-A*01	Human cytomegalovirus, 65 kDa phosphoprotein (hCMV, pp65)	363–373
CMV-VIE1	ELRRKMMYM	HLA-B*08	Human cytomegalovirus, 55 kDa immediate-early protein 1 (hCMV, VIE1)	199–207
EBV-GP350	PRPVSRFLGNNSILY	HLA-DR	Epstein-Barr virus, Envelope Glycoprotein GP350 (EBV, gp350)	268–282
CMV-pp65^283–299^	KPGKISHIMLDVAFTSH	HLA-DR	Human cytomegalovirus, 65 kDa phosphoprotein (hCMV, pp65)	283–299
CMV-pp65^510–524^	YQEFFWDANDIYRIF	HLA-DR	Human cytomegalovirus, 65 kDa phosphoprotein (hCMV, pp65)	510–524

### Multi-peptide vaccine

A multi-peptide vaccine was prepared mixing ADV-Hex, FLU-NCAP and EBV-GP350 peptides (Table 1) with XS15 in water/20% DMSO. This vaccine was emulgated 1:1 with Montanide™ ISA51 VG (Seppic, Paris, France) using an established protocol, injecting 400 μl, containing 80 μg XS15 and 240 μg of each peptide *s.c.* abdominally.

The second vaccination 14 months later contained CMV-VPAP, HLA-CMV-pp65, CMV-VIE1, CMV-pp65^283–299^ and CMV-pp65^510–524^ peptides (Table 2). This vaccine was prepared and administered as described, but to a different site in roughly the same lymph collection area as the first vaccination and contained 50 μg XS15 in 400 μl.

### Dendritic cells (DC)^*^

DCs were differentiated from PBMCs, culturing adherent cells with human GM-CSF and IL-4, (both PeproTech, Hamburg, Germany). Cells were either left untreated, matured with a mix of IL-1β, TNF (both PeproTech), PGE2 (Sigma-Aldrich), poly(I:C) and R848 (both InvivoGen), or treated with Pam_3_CysSK_4_ or XS15.

### Immunomagnetic isolation of slanMo, NK cells, and CD4^+^ T cells^*^

Isolation of slanMo was performed as described previously [21]. PBMCs were incubated with M-DC8 antibody containing hybridoma supernatant, labeled with rat anti-mouse IgM coupled to paramagnetic microbeads (Miltenyi Biotec, Bergisch-Gladbach, Germany) and sorted (autoMACS; Miltenyi).

CD56^+^ CD3^neg^ NK cells and CD3^+^ CD4^+^ T cells were isolated from PBMCs by immunomagnetic depletion (Miltenyi). Purity of sorted cells > 90% was confirmed by flow cytometry.

### Flow cytometry^*^

DCs were stained with CD14-Alexa Fluor 700 (eBioscience, San Diego, CA), CD83-APC and CD86-BV605 (Biolegend), HLA-DR-PerCP, TLR2-PE (BD Biosciences, Heidelberg, Germany) and Zombie Aqua (Biolegend) after Fc Block (BD), fixed and measured (LSR Fortessa; BD Biosciences).

Surface molecules of slanMo, NK cells, and CD4^+^ T cells were characterized with CD3-FITC, CD4-PE, CD56-PE, HLA-DR-APC (all BD), and M-DC8 hybridoma supernatant [21] to determine their purity (FACSCalibur; BD).

For intracytoplasmic staining of IFNγ and IL-4, CD4^+^ T cells were stimulated in the presence of phorbol myristate acetate (PMA) and ionomycin (both from Sigma-Aldrich) and brefeldin A was added. IFNγ-FITC and IL-4-PE (both from BD) staining was performed and analysed.

### Cytokine assay^*^

slanMo were maintained allowing spontaneous maturation into DCs and cultured in the presence of XS15 or XS15 + IFNγ to stimulate cytokine secretion. TNF, IL-1β, IL-6, IL-12, and IL-23 were determined by ELISA (BD) in supernatants. Further, matured slanMo were coincubated with the CD8^+^ T cell clone CC7 [22], in the presence of the pertinent recognized WT1 peptide RMFPNAPYL + XS15, quantifying IFNγ in supernatants. Likewise matured slanMo were coincubated with autologous NK cells and IFNγ quantified.

### T-cell programming^*^

Matured slanMo were cocultured with allogeneic CD4^+^ T cells and XS15. Harvested T cells were incubated with PMA/ionomycin. Cells were analysed for IFNγ and IL-4 production by flow cytometry.

### Statistical analysis

Results were assessed by Student’s *t*-test or Analysis of Variance (ANOVA), with *p* ≤ 0.05 considered significant.

### Human B and NK cell activation^*^

Fresh PBMCs were cultured either alone, with Pam_3_CysSK_4_ or XS15, or a mix of phytohaemagglutinin-L (PHA) and Pokeweed mitogen (PWM). Adherent and non-adherent cells were stained with mAbs: CD3-BV711, CD56-BV421, CD19-BV785, Zombie aqua (all Biolegend), CD14-Alexa Fluor 700, HLA-DR-PerCP, and CD69-APC-Cy7 (all BD). Cells were measured by flow cytometry as described above.

### Isolation of cells from the granuloma^*^

After surgical removal of the vaccine-induced granuloma, tissue was used for in vitro expansion of granuloma infiltrating T cells (GICs) after dissociation by combined mechanical and enzymatic processes, filtering (100 μm) and separation over a density gradient. Isolated cells were phenotyped and measured (ELISpot assay).

### Phenotyping of granuloma infiltrating T cells (GICs)^*^

GICs and PBMCs were stained for CD3-PE-Cy5.5 (eBioscience), CD4-BV711, CD8-PerCP, CD25-BV605, CD45RA-BV570, CCR7-BV650, CD39-BV421, PD-1-APC-Cy, BTLA-PE, Tim-3-PE-Cy7 (all Biolegend), LAG3 (Enzo Life Sciences, Lörrach, Germany), CTLA4-PE-CF594 (BD) and Live/dead-Aqua dye (Life technologies, Carlsbad, CA) or with isotype controls. Cells were fixed and permeabilized, followed by ICS using Foxp3-FITC (eBioscience) and Ki67-Alexa Fluor 700 (BD) and measured on an LSR Fortessa (BD).

### In vitro expansion of GICs^*^

Granuloma tissue pieces were cultured and expanded for 12 days in specialized TIL culture medium containing IL-2 and anti-CD3 antibody (clone OKT3, Miltenyi).

### IFNγ ELISpot^*^

IFNγ secretion by PBMCs and GICs in response to peptide stimulation was determined using ELISpot assay [23].

### Multimer staining^*^

Multimer staining conformed essentially to the protocol suggested by CIP (http://www.cimt.eu/workgroups/cip) as outlined previously [6].

### Estimation of functional vaccine-specific T cells in the granuloma and in peripheral blood^*^

For a rough estimate of vaccine-specific T cells, respective cells within the granuloma were calculated based on experimental results (see Additional file 1: *Supplementary Materials and Methods*).

### Degranulation and intracellular cytokine staining (ICS)^*^

ICS was performed as reported earlier [23]. Cells were stimulated with individual peptides or with an equal volume of water/ 10% DMSO in the presence of anti-CD107a (BD), GolgiStop (BD) and Brefeldin A (Sigma-Aldrich). After 12 h cells were stained for CD4-APC-Cy7 (BD), CD8-PECy7 (Beckman Coulter) and CD3-BV711 (Biolegend) and with Aqua Live Dead, fixed and permeabilized in (Cytoperm/Cytofix; BD) and further stained for IFNγ-Alexa Fluor 700 (BD Biosciences), anti-TNF-Pacific Blue (Biolegend), IL-10-PE and IL-2-APC (both BD).

### Luminex multiplexed bead-based sandwich immunoassay^*^

Levels of 42 proteins and immune-associated markers were measured using the Luminex 100/200 instrument. The kit components and software for data analysis of the multiplexed immunoassay were kindly provided by Myriad RBM, Austin, TX (http://rbm.myriad.com) and used as specified. Serum samples were tested in singles.

### Detection of antibody responses against the vaccinated peptides and XS15^*^

Antibodies were detected by ELISA with an in-house assay as published previously [24]. XS15 coated microtiter-plates were incubated with sera from the vaccinated individual as well as pertinent controls. Bound antibodies were detected with peroxidase conjugated goat anti-human IgG- and IgM-antibodies (DIANOVA, Hamburg, Germany).

### Isolation of HLA ligands from granuloma tissue and detection of vaccinated peptides by mass spectrometry^*^

HLA class I and HLA-DR ligands were isolated by immunoaffinity purification from granuloma tissue with W6/32 and L243 antibodies (both produced in-house) as described previously [25]. HLA ligand extracts were analysed by tandem mass spectrometry (LC-MS/MS) using an Orbitrap Fusion Lumos and Ultimate3000 RSLCnano system (both ThermoFisher Scientific). Data processing was performed by SEQUEST database search against the reviewed Swiss-Prot human reference proteome concatenated with the vaccinated peptide sequences, verifying identifications by comparison with fragmentation patterns of isotope-labeled peptides of identical sequence.

### Transcriptome sequencing and analysis of differential gene expression^*^

RNASeq was performed by an external service provider (CeGaT, Tübingen, Germany). RNA was isolated from the granuloma center & margin and distal edge. Single end sequencing was performed (HiSeq 2500; Illumina San Diego CA). Mapping (hg19) (STAR software, V. 2.4.0), data processing and counts of mapped reads were computed (Cufflinks Tool Suite; Version 2.1.1). FPKM values were calculated (Cuffdiff) employing a pooled-variance model and geometric normalization with multi-read-correction (Additional files 2, 3, 4). Differential gene expression (FC > 5, q < 0.05) in the granuloma center vs. the margin was assessed (Additional file 5) and a pre-selected gene set of interest (hallmark inflammatory response gene set, comprising 200 genes; last accessed: December 2018; http://software.broadinstitute.org/gsea/msigdb/cards/HALLMARK_INFLAMMATORY_RESPONSE.html) compared for the three different tissue regions sampled (Additional file 6).

### Histology and immunohistochemistry^*^

A tissue sample of the granuloma center was processed as formalin-fixed paraffin embedded (FFPE) tissue, cut into 3–5 μm sections and stained by HE for histological evaluation. Granulocytes were identified by typical appearance as well as mineral oil deposits (representing vaccine remnants) appearing as large vacuolar structures. Immunohistochemistry staining was performed (BOND-MAX, Leica Biosystems, Wetzlar, Germany) with monoclonal antibodies recognizing CD8, CD68, CD20 (all Dako, Glostrup Denmark) and CD4 (Cell Marque, Rocklin, CA). Appropriate positive and negative controls were included.

### Immunofluorescence staining of slanMo and CD8^+^ T cells^*^

FFPE tissue sections were deparaffinized in xylene, hydrated by washes of graded ethanol to water and boiled in citrate buffer. Tissues sections were stained with mouse anti-CD8 antibodies (Dako) and the mouse anti-slan antibody DD2 (in-house, Institute of Immunology, Medical Faculty Carl Gustav Carus, Dresden). CD8^+^ T cells were visualized by an AF633-labeled goat anti-mouse IgG antibody (ThermoFisher Scientific) and slanMo by a goat anti-mouse IgM Biotin (1:100, Southern Biotech, Birmingham, AL), followed by AF546-labeled Streptavidin (ThermoFisher Scientific). Tissues were mounted on DAPI-containing AKLIDES® ANA plus medium (Medipan, Dahlewitz, Germany), coverslipped and evaluated (BZ-9000; Keyence, Osaka, Japan). For quantification of slanMo and CD8^+^ T cells, positively stained cells were counted in 15 different high power fields (HPF) of a tissue section using the Vectra imaging platform (Akoya Biosciences, Hopkinton, MA, USA) and the mean value was determined. The mean number of cells per HPF (area: 0.3345mm^2^) was converted to square millimeter.

### ^18^F-FDG PET/MR scan

To investigate the injection site and draining lymphoid organs, a dynamic positron-emission tomography (PET)/ magnetic resonance tomography (MR) scan of the abdomen was performed after injection of 209 MBq ^18^F-2-Fluor-2-desoxy-D-glucose (^18^F-FDG; *i.v.*) using a 3 T-PET/MR scanner (Biograph mMR, Siemens Healthineers, Erlangen, Germany). PET was reconstructed with an OSEM-3D algorithm, applying a MR-based attenuation map. For morphologic analysis, a T2 Half-Fourier Acquisition Single-shot Turbo spin Echo (HASTE) and a T2 Turbo inversion recovery magnitude TIRM sequence was assessed.

## Results

### Design of Pam_3_Cys-GDPKHPKSF (XS15)

**Fig. 1:**
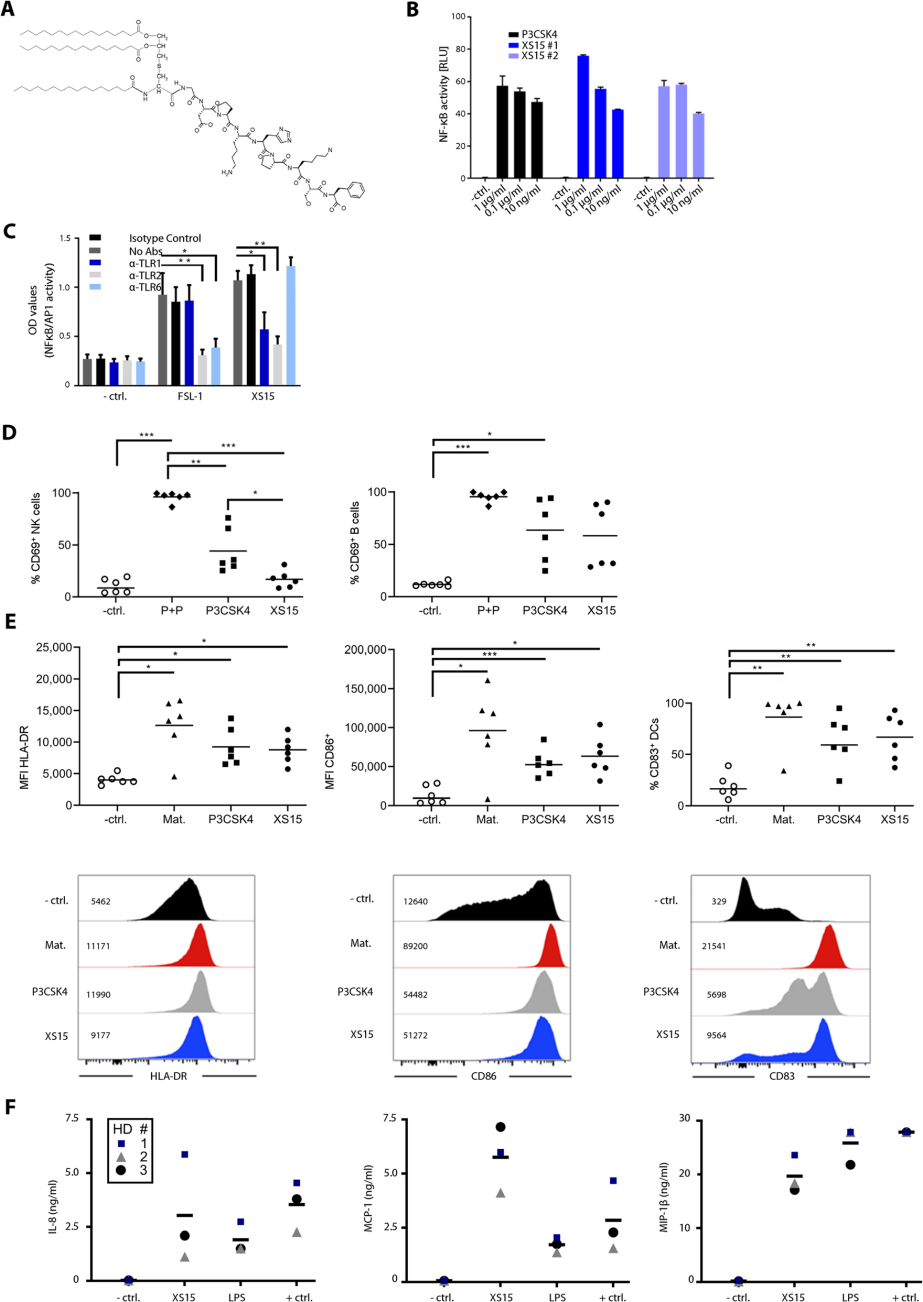
Pam_3_Cys-GDPKHPKSF (XS15) is a TLR1/2 ligand activating immune cells and stimulating DCs and cytokine release**.** (**a**) Structure of Pam_3_Cys-GDPKHPKSF: Skeletal structural formula of the molecular structure of the lipopeptide Pam_3_Cys-GDPKHPKSF termed XS15. (**b**) Dual-luciferase assay on HEK293T cells transfected with TLR2: HEK293T cells were transiently transfected with a human TLR2 plasmid and a NF-κB luciferase reporter plasmid or left untreated (− ctrl.). Culture medium was replaced after 30 h and stimuli added at the stated concentrations. The cells were incubated for 18 h and lysates were prepared and analysed by dual-luciferase assay. Pam_3_CysSK_4_ (P3CSK4) and two different lots of XS15 (XS15#1/ XS15#2) were used. (**c**) HEK-Dual hTLR2 cells, stably expressing a NF-κB/AP-1-inducible secreted embryonic alkaline phosphatase (SEAP) reporter, were incubated for 1 h with TLR1, TLR2 and TLR6 blocking antibodies, isotype control or negative controls (no Abs) (4 μg/ml). Then, cells were stimulated for 24 h with the established TLR2/6 agonist FSL-1 (1 ng/ml), XS15 (10 ng/ml) or left unstimulated (− ctrl.). Supernatants were collected and SEAP levels determined using QUANTI-Blue detection assay. Error bars represent SD. The graph shows the mean + SEM of *n* = 2 experiments, significance was assessed by two-way ANOVA. (**d**) Immune cell activation by XS15: Fresh PBMCs were cultured for 40 h in the presence of Phytohemagglutinin-L (PHA) + Pokeweed (PWM) (P + P), Pam_3_CysSK_4_ (P3CSK4), XS15 or left untreated (− ctrl.). Activated NK (*left panel*) and B cells (*right panel*) were assessed with the marker CD69 following the gating strategy: time gate, single cells (FSC-H/ FSC-A), living cells (Zombie-Aqua/ FSC-A), lymphocytes (FSC-A/ SSC-A); B-cells were defined as CD14^neg^ CD3^neg^ CD19^+^ cells and NK cells as CD14^neg^ CD3^neg^ CD19^neg^ CD56^+^ cells. Healthy donors (*n* = 6), means are shown, significance was assessed by one-way ANOVA. (**e**) Dendritic cell (DC) stimulation by XS15: DCs were differentiated from blood monocytes and then matured as described in the material and methods section. Gating strategy was: time gate, single cells (FSC-H/ FSC-A), living cells (Zombie Aqua/ FSC-A). *Upper panel*: scatter plots for healthy donors (*n* = 6), means are shown significance was assessed by one-way ANOVA. *Lower panel*: modal histograms and median fluorescences for one representative donor. Medium control without maturation cocktail = − ctrl. Standard maturation cocktail = Mat. (**f**) Induction of cytokine release by XS15: Anticoagulated whole blood was incubated with XS15 (10 μg/ml) as well as LPS (100 ng/ml) and PHA (2 μg/ml)/ PWM (1 μg/ml) as positive (+ ctrl.) and medium only as negative controls (− ctrl.) and supernatants harvested after 20 h. Multiplexed bead-based sandwich immunoassays were performed using a LUMINEX device with a 42-analyte panel. Exemplary findings obtained in three healthy donors (HD) for IL-8 (*left*), MCP1 (*middle*) and MIP-1β (*right*) are shown with means. HD1 (blue square) designates the vaccinated volunteer characterized in more detail subsequently. Additional results are provided in Additional file 7: Table S1. In case of saturation, the upper limit of quantification (ULOQ) was assigned. *p* ≤ 0.05*; **p ≤ 0.01; ***p ≤ 0.001

Pam_3_Cys-derivates, such as Pam_3_Cys-SK4 [26], are water-soluble amphiphilic compounds, exhibiting detergent characteristics and can induce unspecific effects at higher concentrations [27]. We therefore designed a new lipopeptide (chemical structure in Fig. 1a) with nearly even charge balance, derived from a naturally occurring sequence (GDPKHPKSF) in *Mycoplasma salivarium* [28]. The compound can be generated in very high purity by conventional chemistry and purification procedures, is water-soluble, can be sterilized by 0.2 μm filtration, and thus is GMP-amenable. This new compound was designated XS15.

### Initial in vitro characterization of XS15

To confirm TLR2 activity, we used HEK cells transiently transfected with TLR2 in an NF-κB reporter system, as established readout to measure TLR2 activity [29]. Dose escalations compared to the standard Pam_3_CysSK_4_ revealed a similar activity of XS15, absent in TLR2-negative HEK cells (Fig. 1b). Since it is established that Pam_3_Cys is a ligand of TLR1/2 heterodimers, also by crystal structure analysis [30], we assumed that XS15 is also a TLR1/2 ligand. This was confirmed by antibody blocking experiments (Fig. 1c). Incubation of PBMCs with XS15 showed CD69 induction on B (*p* = 0.055), but not on NK cells, within 40 h (Fig. 1d), both cell types were reported to show similar TLR2 levels, whereas B cells show increased TLR1 expression [31]. The stimulation of monocyte-derived DCs with XS15 significantly induced HLA-DR, CD83 and CD86, in line with the reported expression of TLR2 on DCs [32] (Fig. 1e). To assess induction of cytokine production, fresh citrate anticoagulated whole blood from three volunteers was incubated with XS15, LPS or PHA/ PWM as positive control. After 20 h, supernatant was harvested and subjected to Luminex multiplexed bead-based sandwich immunoassays. A particularly strong induction of IL-8, MCP1, and MIP-1β was observed, albeit with considerable inter-donor variance as commonly observed in humans [33], indicative of the activation of innate immune cells (Fig. 1f; Additional file 7: Table S1).

### XS15 efficiently augments functional properties of 6-sulfo LacNAc-expressing monocytes

**Fig. 2:**
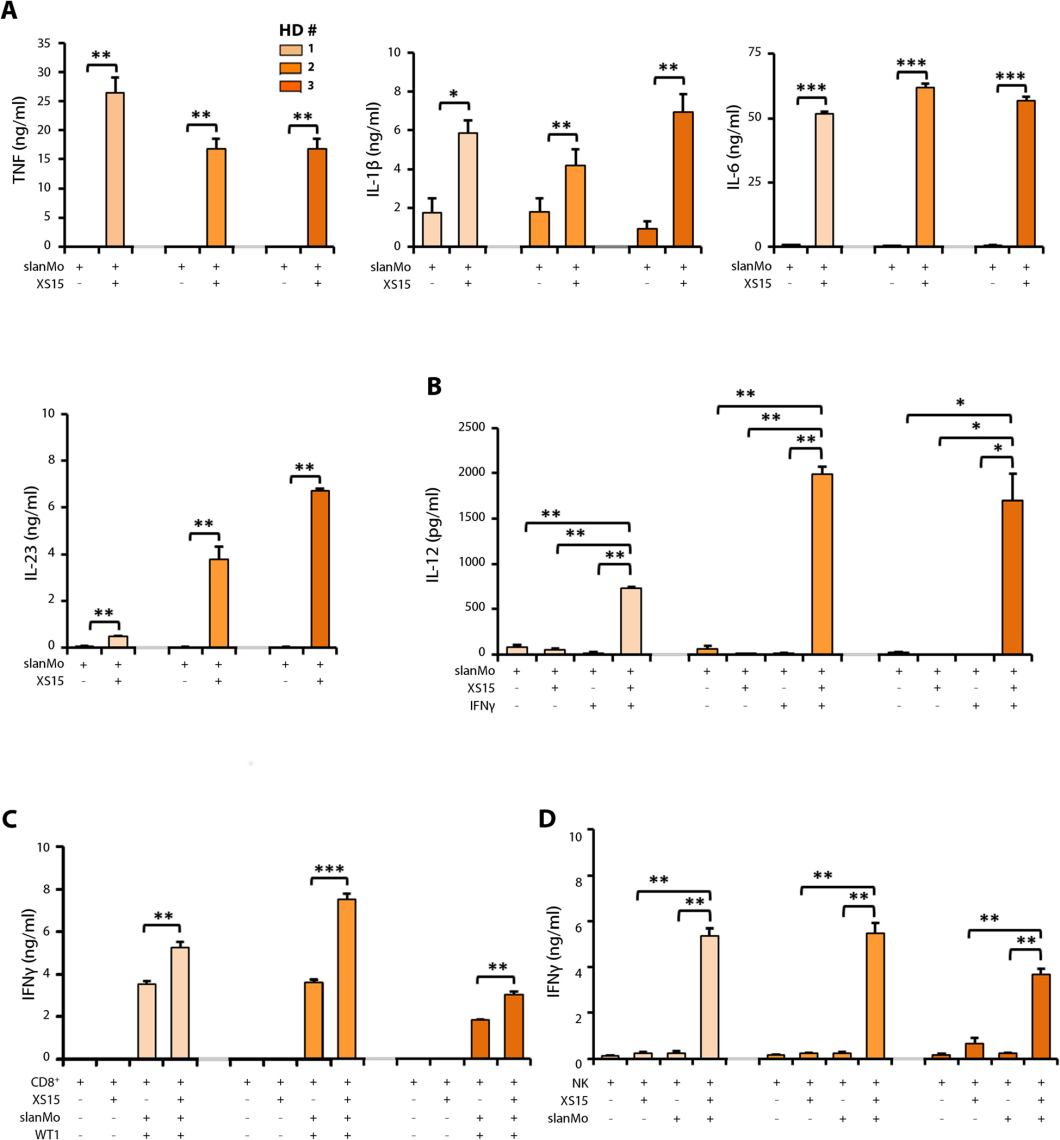
Impact of XS15 on cytokine release by slanMo and their capacity to stimulate WT1 peptide-specific CD8^+^ T cells and NK cells. (**a**) slanMo were maintained for 6 h to allow spontaneous maturation into DCs. Subsequently, slanMo were cultivated alone (slanMo) or in the presence of XS15 (slanMo + XS15) for additional 18 h. Supernatants were collected and the concentration of (**a**) TNF (*left*), IL-1β (*middle*), IL-6 (*right*), IL-23 (*lower left*) analysed by ELISA. (**b**) slanMo were maintained for 6 h to allow spontaneous maturation into DCs. Subsequently, slanMo were cultivated in the absence (slanMo) or presence of XS15 (slanMo + XS15) for additional 18 h, alternatively, slanMo were incubated with IFNγ for the first 6 h. Thereafter, slanMo were cultivated in the presence of IFNγ alone (slanMo + IFNγ) or together with XS15 (slanMo + IFNγ + XS15) for additional 18 h. Then, IL-12 was analysed by ELISA. The results of three different healthy donors (HD) are presented as mean ± SE of duplicate or triplicate measurements. (**c**) Effect of XS15 on the capacity of slanMo to stimulate IFNγ release by WT1 peptide-specific CD8^+^ T cells: slanMo were maintained for 6 h to allow spontaneous maturation. Subsequently, slanMo were coincubated with the specific CD8^+^ T cell clone CC7 (slanMo + CD8^+^), in the presence of the WT1 peptide (WT1) and/or XS15. After 42 h, supernatants were collected and IFNγ was quantified by ELISA. The results of three different healthy donors (HD) are presented as mean ± SE of triplicate determinations. (**d**) Impact of XS15 on the ability of slanMo to stimulate IFNγ secretion by NK cells: slanMo were maintained for 6 h to allow spontaneous maturation. Then, autologous NK cells were cultured either alone (NK) or incubated with XS15 (NK + XS15), cocultured with slanMo alone (NK + slanMo) or additionally incubated with XS15 (NK + slanMo +XS15). After 42 h, supernatants were collected and the concentration of IFNγ was determined by ELISA. The results of three different HD are presented as mean ± SE of triplicate determinations. Asterisks indicate a statistically significant difference (*p ≤ 0.05; **p ≤ 0.01; ***p ≤ 0.001; assessed by Student’s *t*-test). Exemplary flow cytometry results showing effects of XS15 on slanMo-mediated T cell programming regarding the percentage of IFNγ- and IL-4-producing CD4^+^ T cells are provided as Additional file 8: Fig. S1

6-sulfo LacNAc^+^ monocytes (slanMo, formerly termed M-DC8^+^ DCs or slanDCs) represent a particularly proinflammatory subset of human non-classical blood monocytes that can undergo a differentiation process into DCs [21, 34–36]. Previously, we have demonstrated that slanMo display prominent expression of TLR2 and produce large amounts of various proinflammatory cytokines upon activation with TLR2 agonists [21, 34]. Further studies revealed that slanMo efficiently activate T lymphocytes and NK cells [21, 36, 37]. Based on these proinflammatory features of slanMo, we explored the impact of XS15 on various immunostimulatory properties of this cell subset. To investigate the influence of XS15 on their cytokine release, slanMo were maintained for 6 h to allow spontaneous maturation into DCs and cultured in the presence of XS15 subsequently. XS15 efficiently enhanced the capacity of slanMo to secrete the proinflammatory cytokines TNF, IL-1β, IL-6, and IL-23 (Fig. 2a), whereas IL-12 production was not influenced. Interestingly, combined XS15 and IFNγ significantly augmented IL-12 release by slanMo (Fig. 2b).

Further, the impact of XS15 on the ability of slanMo to promote T helper (T_H_)-programming was explored. Therefore, slanMo were coincubated with allogeneic CD4^+^ T cells in the presence of XS15. Notably, XS15 markedly enhanced the capability of slanMo to favor the differentiation of CD4^+^ T cells into IFNγ-producing T_H_1 cells (Additional file 8: Fig. S1). In contrast, the ability of slanMo to polarize CD4^+^ T cells into IL-4-expressing T_H_2 cells was not modulated by XS15. To investigate whether XS15 enhances the capacity of slanMo to activate antigen-specific CD8^+^ T cells, Wilms’ tumor antigen 1 (WT1) peptide-loaded slanMo were coincubated with XS15 and the WT1 peptide-specific CD8^+^ T cell clone CC7 [38]. XS15 significantly augmented the capacity of slanMo to stimulate IFNγ secretion by WT1 peptide-specific CD8^+^ T cells (Fig. 2c). To exclude a potential contribution of slanMo to the IFNγ content of the supernatants derived from the slanMo-T cell coculture, we determined the intracellular IFNγ expression by flow cytometric analysis. XS15-activated slanMo did not express IFNγ (Additional File 8: Fig. S2).

Furthermore, the influence of XS15 on slanMo mediated NK cell activation was evaluated. Coculture of slanMo with autologous NK cells in the presence of XS15 significantly enhanced the ability of slanMo to stimulate IFNγ release by NK cells (Fig. 2d).

### XS15 is an effective vaccine adjuvant

**Fig. 3:**
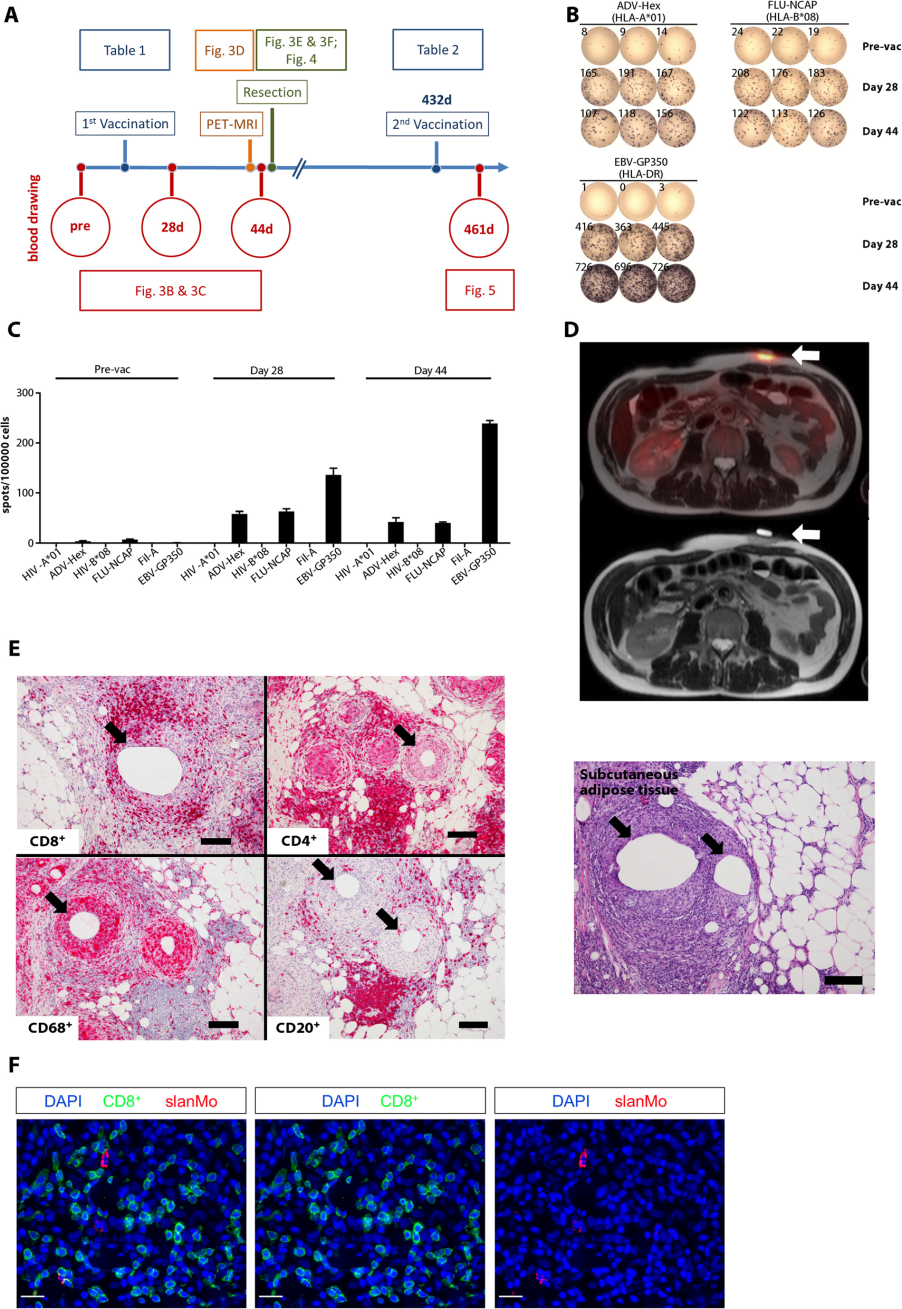
A single vaccination with peptides and XS15 induces a granuloma and local immune cell infiltration with functional T cells. (**a**) Time line providing an overview on blood and tissues samples as well as analyses described subsequently and interventions performed (i.e. vaccination, ^18^F-FDG-PET-MR imaging/ granuloma resection). Vaccinated peptides used at each time point are provided in Tables 1 & 2, respectively; Pre (before vaccination); d (day after first vaccination). (**b**) Induction of functional T cells by XS15 detected in ex vivo ELISpot: PBMCs were isolated from peripheral blood of a volunteer before vaccination (pre-vac), 28 days and 44 days after vaccination. IFNγ response towards the three vaccine peptides (ADV-Hex, FLU-NCAP and EBV-GP350) was determined in two ELISpot assays (Pre-vac + 28d, and 44d). HIV-A*01, HIV-B*08 and Fil-A peptides served as the relevant negative controls. 300,000 cells were seeded per well. Phytohemagglutinin-L (PHA-L) stimulation was used as a positive control (ELISpot plate wells rearranged, and negative controls left out). (**c**) Respective mean spot counts and SD/100,000 cells per well are shown. (**d**) Granuloma formation at the vaccination site: ^18^F-FDG-PET/MR (upper panel) performed on day 43 demonstrated an intense ^18^F-FDG uptake at the site of the induration (standardized uptake value ((SUV(mean) 4.6; SUV(max) 6.4), but no ^18^F-FDG-uptake was observed in the draining lymph nodes or in any other secondary lymphoid organs; corresponding MR (lower panel). (**e**) Immune cell infiltration of the granuloma induced by vaccination: A tissue sample from the granuloma center was processed as formalin-fixed paraffin-embedded (FFPE) tissue and assessed by hematoxylin & eosin (HE) staining (right) and immunohistochemistry (left). T cells (CD8^+^ and CD4^+^), B cells (CD20^+^) as well as macrophages (CD68^+^) and granulocytes appeared as ordered structures in separated areas resembling lymphoid tissues. Mineral oil deposits (black arrows) were still discernible, surrounded by macrophages, whereas both CD4^+^ and CD8^+^ T cells were located closely to the macrophages but separated from the oil patches. Original magnification was × 100. Black scale bars indicate 200 μm. (**f**) Co-localization of slanMo and CD8^+^ T cells in the granuloma. Immunofluorescence staining was performed to detect slanMo and CD8^+^ lymphocytes in the granuloma of the XS15-vaccinated volunteer. As representative examples, images of single CD8^+^ T cell or slanMo stainings as well as merged images are shown. Original magnification was × 400. White scale bars are 20 μm

We assessed whether XS15 might prove as an effective adjuvant with properties similar to CpG, when used combined with Montanide [6, 39]. An HLA-A*01-restricted adenovirus-derived 10 amino acid (AA) peptide (ADV-Hex, LTDLGQNLLY), an HLA-B*08 influenza-derived 9 AA peptide (FLU-NCAP, ELRSRYWAI) and a promiscuous HLA-DR restricted 15 AA EBV peptide (EBV-GP350, PRPVSRFLGNNSILY), dosed at 240 μg/peptide (Table 1), were emulsified in Montanide together with 80 μg of XS15 and injected subcutaneously (*s.c.;* 400 μl) in the lower abdomen of an HLA-matched volunteer. A time line depicting the course of events is provided in Fig. 3a. Ex vivo IFNγ ELISpot assays (300,000 PBMCs/well) obtained at days 28 and 44 after vaccine administration showed strong reactivities against the HLA class I (107–208 spots) and the HLA class II (416–726 spots) peptides (Fig. 3b). Pre-vaccination ELISpots were negative for the HLA class II peptide and weak for both class I peptides (8–24 spots). Such a strong induction of human T cells in vivo has never been evidenced by us before as a result of any other treatment and is therefore unprecedented in our laboratory (Fig. 3c), however it should be noted that a single case report is unable to provide any conclusive evidence. In a vaccination study in prostate carcinoma patients, utilizing peptides emulsified in Montanide with or without additional adjuvants, we did not detect any ex vivo ELISpot responses, not even after four repetitive vaccinations ( [23, 40]; and unpublished own data). In a study in renal cell carcinoma patients using multi-peptide vaccination (*i.d.*) and GM-CSF, T cell responses against viral or tumor antigens could only be detected after in vitro restimulation [5]. Since ex vivo ELISpot is considered to reflect the activity and quantity of effector T cells, we conclude that the massive induction of functional T cells in this volunteer is best explained by the peptide vaccination with XS15. The individual’s serum was also tested for antibody responses against the vaccine components (days 28, 44, 70, and 119 after the first vaccination). A vigorous induction of antibodies against the vaccine peptides was not observed. Only a moderate IgM induction, but no other antibody class, was observed against XS15 and/or the attached peptide GDPKHPKSF (Additional File 7: Table S2).

### Characteristics of the vaccine-induced granuloma

As expectable with Montanide, a painless granuloma formed at the injection site. The volume increased to about 8 ml, as measured by ultrasound (days 17 and 41), without any sonographic signs of infection. After 21 days it appeared as a well-palpable induration of about 2 × 2 cm, with a central reddish surface. The granuloma was described as not touch-sensitive, whereas the skin surface as slightly itching. Since the PBMCs showed a strong and functional T cell response at day 28, we assessed its metabolic activity and performed a simultaneous PET/MR scan after injection of the glucose analogue ^18^F-2-Fluor-2-desoxy-D-glucose (^18^F-FDG) on day 43. An intense ^18^F-FDG uptake at the site of the granuloma was observed (standardized uptake value (SUV(mean)) 4.6; SUV(max) 6.4), obviously caused by the massive inflammatory response (Fig. 3d). No ^18^F-FDG-uptake was observed in the draining lymph nodes or any other secondary lymphoid organs. Since granulomas caused by Montanide with or without adjuvant may sequester T cells and induce their dysfunction and deletion in mice [7], we aimed to test whether this is reproduced in humans, and therefore surgically removed the granuloma at day 44. FFPE tissue samples from the granuloma center showed T cells (CD8^+^ and CD4^+^) as well as macrophages (CD68^+^), B cells (CD20^+^) and granulocytes appearing as ordered structures in separated areas, resembling lymphoid tissues. Mineral oil deposits (Fig. 3e, black arrows) were still discernible, surrounded by macrophages, whereas both CD4^+^ and CD8^+^ T cells were located closely to macrophages but separated from the oil patches. In line with our findings that XS15 efficiently enhances important immunostimulatory properties of slanMo, granuloma-infiltrating slanMo were detectable (18.9 slanMo/mm^2^) and can co-localize with CD8^+^ T lymphocytes (461.8 CD8^+^ T cells/mm^2^) as demonstrated in (Fig. 3f).

### Immune features of the granuloma

**Fig. 4:**
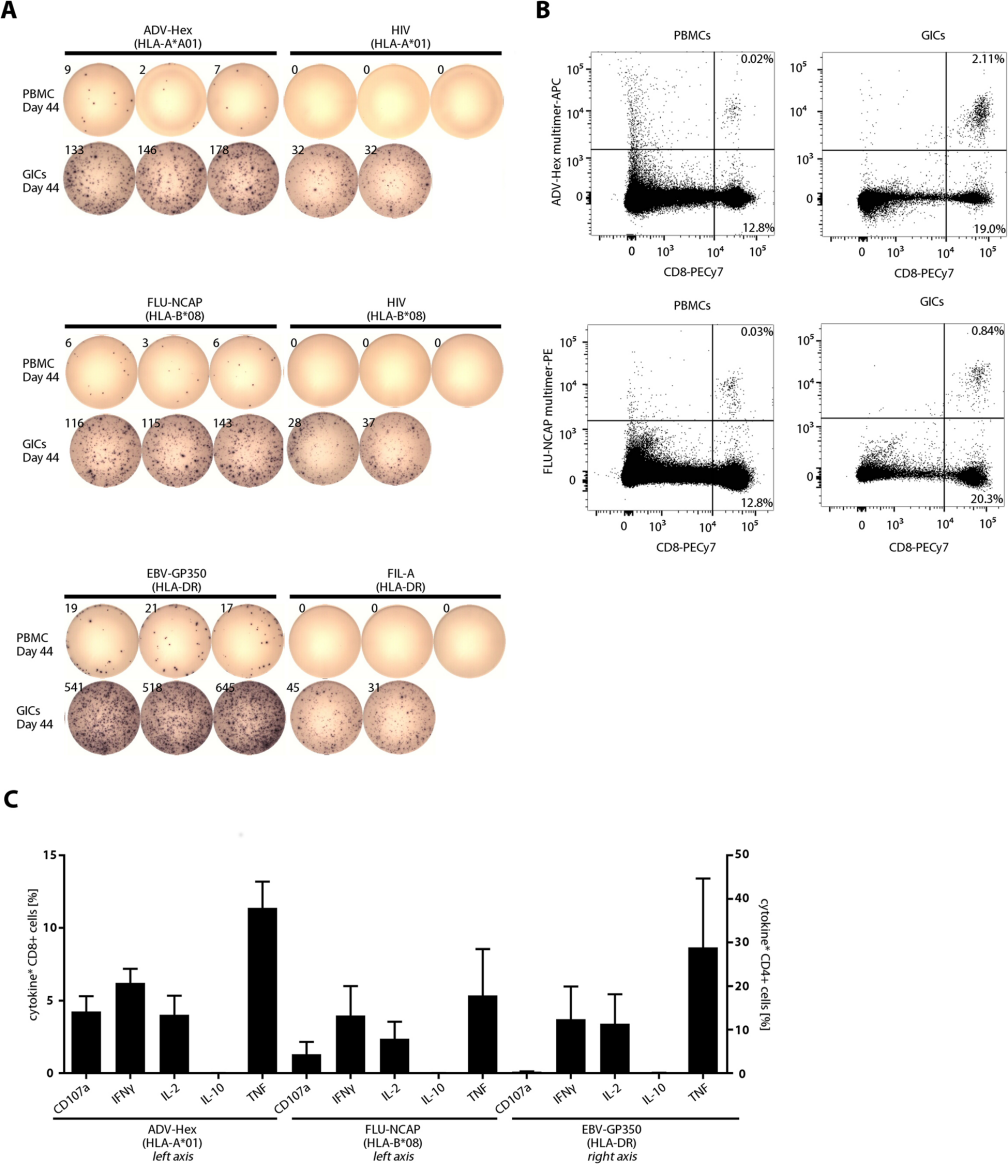
Functionality and antigen-specificity of granuloma infiltrating cells (GICs). GICs were isolated as described in the *Material and Methods* section and analysed alongside PBMCs isolated from blood drawn on the same day from the same individual. (**a**) GICs were rested overnight after isolation and the IFNγ response towards the three vaccinated peptides (ADV-Hex, FLU-NCAP and EBV-GP350; Table 1) was determined by IFNγ ELISpot assay. 50,000 cells were seeded per well. HIV-A*01, HIV-B*08 and Fil-A peptides served as the relevant negative controls (rearranged wells). Ex vivo phenotype of GICs is provided as Additional File 8: Fig. S3. (**b**) PBMCs and GICs were harvested from the ELISpot plate (see panel A) and stained with ADV-Hex APC- and FLU-NCAP-PE- multimers. Percentages of CD8^+^ multimer-positive and multimer-negative cells within CD4^neg^ are indicated. (**c**) GICs were stimulated and expanded in vitro using anti-CD3 mAb and IL-2. The cells were then re-stimulated with the indicated peptides or with an equal volume of 10% DMSO for 12 h and the indicated secreted cytokines and surface CD107a expression (degranulation) were quantified by flow cytometry (% of functional cells are given after subtraction of marker-positive cells in the DMSO control well)

A single cell suspension was prepared from fresh tissue in the center of the granuloma. The GICs consisted of B, T and NK cells, monocytes and granulocytes. Both CD8^+^ and CD4^+^ T cells expressed activation markers (CD25) and proliferated (intracellular Ki67). The majority was of the effector memory phenotype, with much higher frequencies than in PBMCs obtained at the same day (Additional File 8: Fig. S3). The frequency of regulatory T cells (T_reg_; Foxp3^+^CD25^+^) among CD4^+^ cells was similar in the PBMCs vs. GICs (approx. 11%) (Additional file 8: Fig. S4), in addition different checkpoint receptors were characterized in both cell subsets (Additional File 8: Fig. S5). Ex vivo IFNγ ELISpot of GICs (/50.000 cells) revealed an average of 152 and 125 specific spot counts for the HLA class I peptides (ADV-Hex and FLU-NCAP, respectively) and 568 spots for the HLA class II peptide (GP350), with a background of approx. 32 spots, likely due to remnant vaccine peptides on antigen presenting cells within the granuloma (Fig. 4a). This notion was supported by mass spectrometric detection of all vaccinated peptides in HLA ligand extracts purified from the granuloma core (Additional File 8: Fig. S6). Vaccine-specific T cells among GICs and PBMCs were stained by relevant HLA class I peptide-MHC multimers (Fig. 4b); moreover, they were characterized to be multifunctional after in vitro expansion, confirmed by production of IFNγ, TNF, IL-2 and CD107a, but not IL-10 (Fig. 4c). The total number of vaccine-antigen specific functional T cells was estimated at 3.0 × 10^5^ in the granuloma and 20.5 × 10^6^ in peripheral blood. Thus, in contrast to data reported from mice [7], the granuloma evidenced in a human volunteer induced by Montanide, peptide and XS15 did not show features of a destructive sink for the majority of antigen specific T cells.

For gene expression analysis, samples from the granuloma center, margin and distal edge appearing as unaffected skin were analysed by transcriptome sequencing to assess differential gene expression by the vaccination/ XS15 (complete datasets provided in Additional files 2, 3, 4). Overexpression was observed for 320 genes in the granuloma center vs. margin (FC > 5, q < 0.05; Additional file 5). Differential gene expression was evaluated for a pre-selected gene set of interest, revealing an up-regulation of the majority of the immune-related genes in the granuloma center compared to the outer margin (Additional File 6). Of note: 1) In addition to the Pam_3_Cys receptors TLR1 and TLR2, most other TLRs were found upregulated, including TLR7. 2) Several cytokines and cell surface molecules indicative of a CD8^+^/T_H_1 CD4^+^ response were induced, such as IFNγ, CD8, CD4, and CD80. 3) Immunoglobulin IgG1 heavy chain was identified as one of the genes showing the highest expression in the granuloma center and the strongest upregulation compared to the granuloma margin, which is congruent with the massive B cell infiltration observed by histology. 4) HLA genes showed high basal levels, in particular β2-microglobulin, or were strongly induced, which was particularly pronounced for HLA class II genes.

### Massive induction of CMV-specific T cells after single peptide vaccination and long-lasting memory and boosting

**Fig. 5:**
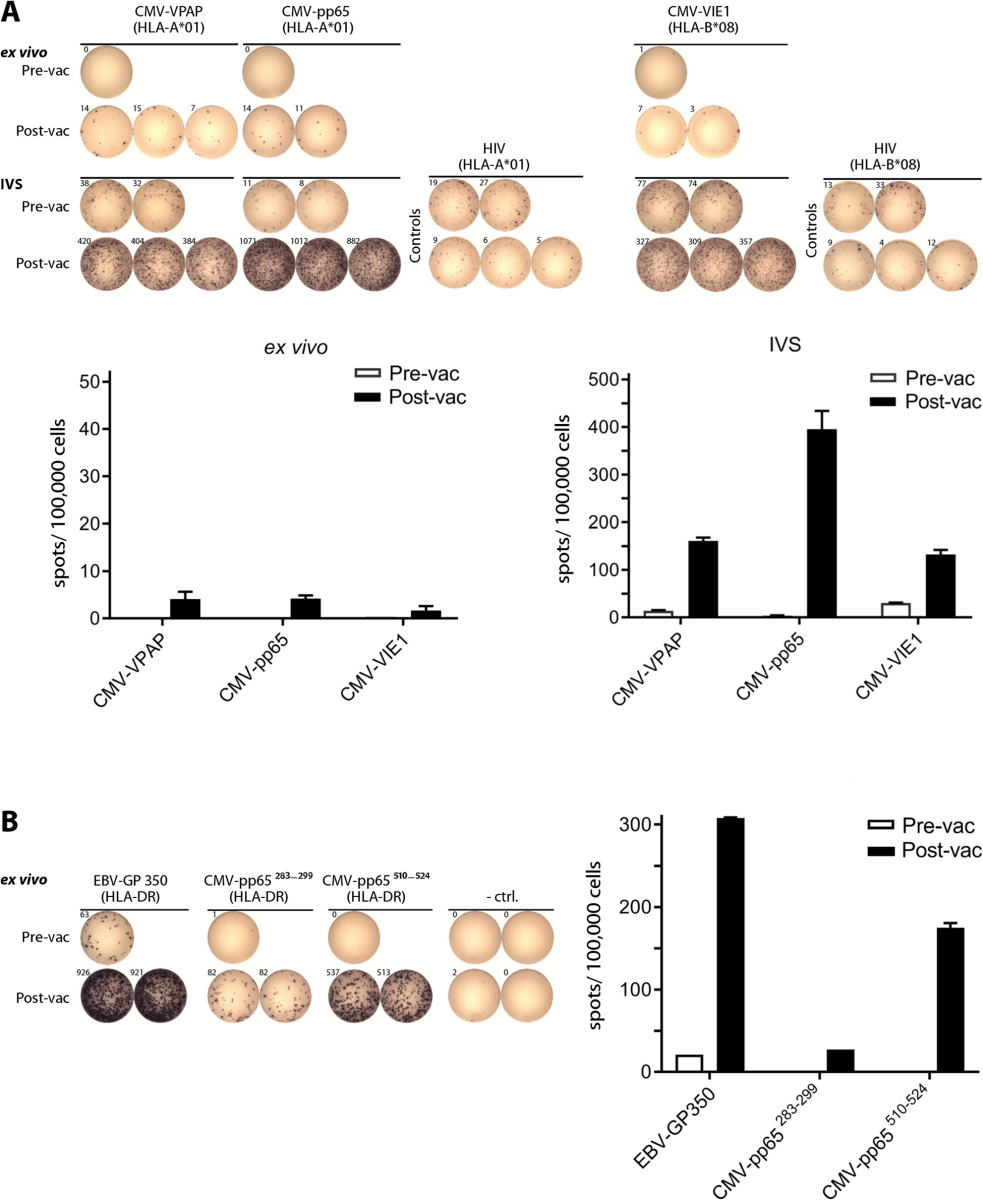
Induction of CMV specific T cells after a single multi-peptide vaccination, and evidence for long lasting memory and boosting. The same volunteer as previously shown was vaccinated with the peptides shown in Table 2, this time with 50 μg of a new batch of XS15. At day 28 after vaccination (Post-vac), PBMCs were assayed by ex vivo ELISpot (**a**; *upper panel* and **b**, 300.000 cells/well), and additionally tested after a short time of in vitro expansion in the presence of the relevant peptides (in vitro *stimulation*; IVS) (A; *lower panels*, 250.000 cells/well). Reactivities against the HLA class I and HLA class II peptides are shown in panels (**a**) and (**b**), respectively (rearranged wells). In addition, bar charts with respective mean spot counts /100,000 cells + SD (when applicable) are shown. Negative control (− ctrl.) was DMSO or respective HLA-matched peptides (HIV); vac (vaccination)

More than one year after the first vaccination, the volunteer (CMV seronegative), was vaccinated with a new multi-peptide cocktail (Tab. 2). The vaccine contained five CMV-derived peptides as well as the EBV-GP350 peptide used already for the first vaccination, now combined with 50 μg of XS15. The HLA class I peptides induced a weak ex vivo T cell response (Fig. 5a; top panel), which increased after a short in vitro pre-sensitization with the respective peptides (Fig. 5a; middle panel). Reactivity against the EBV-GP350 peptide, which had been used in the first vaccination 14 months before, was still detectable ex vivo (approx. 60 spots) before the second vaccination, and increased to more than 900 spots one month after the second vaccination (Fig. 5b), indicating a strong boosting effect. Both newly vaccinated HLA class II CMV peptides stimulated a strong ex vivo T cell response after one single vaccination.

## Discussion

We have evidenced efficient activation of CD4^+^ and CD8^+^ peptide-specific T cells after one single injection of a three-peptide cocktail (containing ADV-Hex, EBV-GP350, FLU-NCAP) emulsified in Montanide and combined with the TLR1/2-ligand adjuvant XS15 in one volunteer.

Several observations are noteworthy: For one peptide (EBV-GP350) there was no measurable immune response before vaccination, and for the other two peptides a pre-existing immune response was enhanced at least 10-fold. T cells were functional and of a T_H_1 profile. The granuloma at the injection site contained functional vaccine-specific T cells, featured lymphoid structures and the induction of inflammatory genes, retaining HLA-presented vaccine peptides ≥7 weeks. More than one year after the first vaccination, the T cell response against the EBV-GP350 peptide was still detectable by ex vivo ELISpot. After a second vaccination of the CMV seronegative volunteer with a new vaccine containing CMV peptides and the EBV-GP350 peptide, the response against this GP350 peptide was strongly boosted, and T cell responses against the CMV peptides were stimulated. The second vaccine induced a painless granuloma of about 10 × 6 mm, which could still be localized after 18 months.

In contrast to previous experience, we speculate several reasons may be responsible for the observed high efficacy of our vaccination approach: 1) a durable local depot of antigen-loaded cells is formed, 2) lymphoid structures are newly assembled with orderly located immune cells, 3) these antigen-specific cells are functional and not exhausted, and 4) antigen-specific T cells are also present in the peripheral blood persisting more than one year later.

We conclude that combination of XS15 and uncoupled peptides could be very useful for peptide vaccination in cancer immunotherapy, where a chosen adjuvant might be easily combined with individually selected peptides. We have shown before in mouse experiments that peptides covalently coupled to Pam_3_Cys-Ser-Ser are more efficient than soluble peptides admixed to Pam_3_Cys-Ser-Ser [41]. Additionally, TLR2 ligands conjugated to human papilloma virus-derived peptides were already shown effective in humans for maturing DCs and for activation of antigen-presenting cells, CD8^+^ and CD4^+^ T cells in an ex vivo skin model [42]. Such covalently coupled peptide-adjuvant conjugates typically require much more extensive purification procedures as compared to free peptides and therefore are difficult, time-consuming and expensive for GMP production. Since the newly designed XS15 worked well in a volunteer with soluble peptides admixed to it, this approach may prove amenable to personalized vaccination approaches. It should be noted however, that this single case report cannot provide any conclusive evidence and does not represent a surrogate for clinical testing.

Obviously, vaccination with Montanide is generally associated with the induction of a local granuloma in humans and therefore seems mainly restricted either to applications in a therapeutic setting e.g., in oncology or for prophylaxis of infection in patients at high risk. On the other side, we show in a volunteer that the vaccination approach with XS15 is able to induce a strong immune response after one single vaccination, due to rapid depot formation and induction of functional target-specific T cells, which would be a considerable advantage of this protocol. Although reports from mouse experiments suggest that vaccinations with Montanide may be counterproductive [7, 43], in this instance functional T cell responses were clearly induced both locally and systemically, including the induction of memory, suggesting there may be no issue in humans with this protocol. Although a peptide vaccine trial in malignant melanoma to characterize the application site after injection of incomplete Freund’s adjuvant showed the induction of dysfunctional CD8^+^ T cells with minimal IFNγ production and T cell retention [44], we did not observe any such results, potentially due to the addition of XS15 to the vaccine. In addition, a one-time application may avoid severe adverse events such as anaphylactic reactions witnessed with repetitive application of e.g. GM-CSF [45].

Since the type and amplitude of the induced T cell response depends critically on adjuvants, there is a great need for effective agents. Most natural TLR ligands prove either unsuitable for GMP production, due to difficulties with synthesis and/or their purification and many more exhibit an unfavorable toxicity profile [46]. Recently, another synthetic TLR1/2 agonist with no structural similarity to natural TLR agonists was identified by an extensive screening program [10]. In mice, this adjuvant showed comparable properties to XS15 in many aspects, for instance regarding cytokine induction. Most interestingly, it was shown that with suitable antigens a complete tumor protection could be reached in 100% of animals, when combined with anti-PD-1 treatment.

We recently showed that personalized peptide vaccination, using peptides actually presented on the patient’s own tumor tissue (as confirmed by MS), is feasible in cancer patients, and immune responses against these peptides can be induced [47]. Here, we used intradermal peptide injections with two adjuvants injected separately. T cell responses were only detectable after several injections. We envision personalized peptide vaccination studies using peptides/Montanide/XS15 for several malignancies based on previously published work [48–50]. We anticipate that the identification of appropriate, personalized, immunological targets together with suitable adjuvants is able to produce T cell immunity, achieving tumor rejection or control of residual disease. Since ICI alone proves insufficient in many malignancies [3], the combination with an efficient vaccination approach will be crucial for overcoming these limitations as shown in mouse models [10].

## Conclusion

As GMP-compliant manufacturing of XS15 is feasible, we expect that regulatory challenges associated with adjuvants can be solved, providing an urgently required tool for vaccine development.

Homing of T cells to the tumor, and the therapeutic efficacy of XS15 will be important aims for future clinical trials. Our vaccine protocol seems easily applicable for clinical implementation and may be ideally combined with existing treatments such as ICI. For enforced T cell anti-tumor activity, also harvesting the vaccine induced T cells from the granuloma seems easily feasible, allowing their expansion for adoptive transfer or the transfer of TCR engineered T cells.

Taken together, we introduce a novel promising vaccine adjuvant that lends itself to clinical development, fulfilling all prerequisites (including regulatory requirements) to be of particular interest for future (personalized) peptide vaccination trials.

## Supplementary information

**Additional file 1.** Supplementary Materials and Methods.

**Additional file 2.** Transcriptome Granuloma Center (FPKM values).

**Additional file 3.** Transcriptome Granuloma Margin (FPKM values).

**Additional file 4.** Transcriptome Granuloma Distal Edge (FPKM values).

**Additional file 5.** Gene expression relative Granuloma Center vs. Margin.

**Additional file 6.** Gene expression analysis for gene set HALLMARK_INFLAMMATORY_RESPONSE

**Additional file 7: Table S1.** Induction of cytokine release by XS15. **Table S2.** Antibody responses to XS15.

**Additional file 8: Fig. S1.** Impact of XS15 on slanMo-mediated T-cell polarization. **Fig. S2.** Impact of XS15 on slanMo-mediated IFNγ expression by WT1 peptide-specific CD8^+^ T cells. **Fig. S3.** Ex vivo phenotype of granuloma infiltrating cells (GICs). **Fig. S4.** Ex vivo assessment of regulatory T cells (Treg) in granuloma infiltrating cells (GICs) and PBMCs. **Fig. S5.** Ex vivo phenotype of checkpoint receptors in granuloma infiltrating cells (GICs) and PBMCs. **Fig. S6.** Detection of vaccinated peptides in granuloma tissue by mass spectrometry.

## Data Availability

All data generated or analysed during this study are included in this published article and its supplementary information files.

## Abbreviations

^18^F-FDG: ^18^F-2-Fluor-2-desoxy-D-glucose
AA: Amino Acid
ADV: Adenovirus
APC: Allophycocyanin
BV: Brilliant Violet
CIP: *CIMT* (Association for Cancer Immunotherapy) Immunoguiding Program
*CMV*: *Cytomegalovirus*
CTLA4: Cytotoxic T-lymphocyte-associated Protein 4
Cy: Cyanine
DAPI: 4′,6-Diamidin-2-phenylindol
*DC*: *dendritic cell*
DMSO: Dimethyl Sulfoxide
EBV: Epstein–Barr Virus
ELISA: Enzyme-linked Immunosorbent Assay
ELISpot: Enzyme-linked Immuno Spot Assay
FFPE: Formalin-fixed Paraffin Embedded
Fil-A: Filamin A
FITC: Fluorescein Isothiocyanate
FLU: Influenza
Foxp3: Forkhead-Box-Protein P3
GIC: Granuloma infiltrating T cell
GM-CSF: Granulocyte Macrophage Colony-stimulating Factor
GMP: Good Manufacturing Practice
HD: Healthy Donor
HE: Hematoxylin & Eosin
HEK: Human Embryonic Kidney
HIV: Human Immunodeficiency Virus
HLA: Human Leucocyte Antigen
i.d: Intradermal
ICI: Immune Checkpoint Inhibition
ICS: Intracellular Cytokine Staining
IFN: Interferon
Ig: Immunglobulin
IL: Interleukin
LacNAc: N-Acetyl-D-Lactosamine
LC-MS/MS: Tandem Mass Spectrometry
MCP1: Monocyte Chemoattractant Protein 1
MIP-1β: Macrophage Inflammatory Protein 1 beta
MR: Magnetic Resonance Tomography
NF-κB: Nuclear Factor ‘kappa-light-chain-enhancer’ of activated B-cells
NK: Natural Killer
PBMCs: Peripheral Blood Mononucelar Cells
PE: Phycoerythrin
PET: Positron-emission Tomography
PGE: Prostaglandin E
PHA: Phytohaemagglutinin-L
PMA: Phorbol Myristate Acetate
PWM: Pokeweed Mitogen
RNA Seq: Ribonucleic Acid Sequencing
RNA: Ribonucleic Acid
SD: Standard Deviation
SE: Standard Error
SEAP: *Secreted Embryonic Alkaline Phosphatase*
slanMo: 6-sulfo LacNAc^+^ (N-Acetyl-D-Lactosamine) Monocytes
SUV: Standardized Uptake Value
T_H_: T helper
TIL: Tumor-infiltrating Leucocyte
TLR: Toll-like Receptor
TNF: Tumor necrosis factor
T_reg_: regulatory T cells
ULOQ: Upper Limit of Quantification
WT1: Wilms’ Tumor Antigen 1
