# Supplementary material for: A new synthetic toll-like receptor 1/2 ligand is an efficient adjuvant for peptide vaccination in a human volunteer
Source: J Immunother Cancer. 2019 Nov 15;7:307. doi: 10.1186/s40425-019-0796-5 (PMC6858783; doi:10.1186/s40425-019-0796-5)
Supplement: Supplementary file 1 — Additional file 1. Supplementary Materials and Methods. [file 40425_2019_796_MOESM1_ESM.docx]

A new synthetic toll-like receptor 1/2 ligand is an efficient adjuvant for peptide vaccination in a human volunteer

Hans-Georg Rammensee ^1, 2, 3^, Karl-Heinz Wiesmüller ^4^, P. Anoop Chandran ^1^, Henning Zelba ^1^, Elisa Rusch ^1^, Cécile Gouttefangeas ^1, 2 ,3^, Daniel J. Kowalewski ^1, 5^, Moreno Di Marco ^1^, Sebastian P. Haen ^1, 2, 6^, Juliane S. Walz ^1, 2, 3, 6^, Yamel Cardona Gloria ^1^, Johanna Bödder ^1^, Jill-Marie Schertel ^7^, Antje Tunger ^7, 8^, Luise Müller ^7^, Maximilian Kießler ^7^, Rebekka Wehner ^7, 8, 9^, Marc Schmitz ^7, 8, 9^, Meike Jakobi ^10^, Nicole  Schneiderhan-Marra ^10^, Reinhild Klein ^6^, Karoline Laske ^1^, Kerstin Artzner ^1^, Linus Backert ^1,5^, Heiko Schuster ^1, 5^, Johannes Schwenck ^3, 11, 12^, Alexander N. R. Weber ^1, 3^, Bernd J. Pichler ^3, 12^, Manfred Kneilling ^3, 12, 13^, Christian la Fougère ^2, 3, 11^, Stephan Forchhammer ^13^,Gisela Metzler ^13^, Jürgen Bauer^13^, Benjamin Weide^13^, Wilfried Schippert^13^, Stefan Stevanović ^1, 2, 3^, and Markus W. Löffler ^1, 2, 3, 14, 15^

*^1^Department of Immunology, Institute for Cell Biology, University of Tübingen, Tübingen, Germany.*

*^2^German Cancer Consortium (DKTK) and German Cancer Research Center (DKFZ) partner site Tübingen, Tübingen, Germany.*

*^3^ Cluster of Excellence iFIT (EXC2180) "Image-Guided and Functionally Instructed Tumor Therapies", University of Tübingen, Germany.*

*^4^EMC microcollections GmbH, Tübingen, Germany.*

*^5^Current address: Immatics Biotechnologies GmbH, Tübingen, Germany.*

*^6^Department of Oncology, Hematology, Immunology, Rheumatology and Pulmonology, University Hospital of Tübingen, Tübingen, Germany.*

*^7^Institute of Immunology, Faculty of Medicine Carl Gustav Carus, Technische Universität Dresden, Dresden, Germany.*

*^8^National Center for Tumor Diseases (NCT), Partner Site Dresden, Germany: German Cancer Research Center (DKFZ), Heidelberg, Germany; Faculty of Medicine and University Hospital Carl Gustav Carus, Technische Universität Dresden, Dresden, Germany and Helmholtz Association/ Helmholtz-Zentrum Dresden-Rossendorf (HZDR), Dresden, Germany.*

*^9^German Cancer Consortium (DKTK), Partner Site Dresden, and German Cancer Research Center (DKFZ), Heidelberg, Germany.*

*^10^NMI Natural and Medical Sciences Institute at the University of Tübingen, Reutlingen, Germany.*

*^11^Department of Nuclear Medicine and Clinical Molecular Imaging, University Hospital of Tübingen, Tübingen, Germany.*

*^12^Werner Siemens Imaging Center, Medical Faculty, University of Tübingen, Tübingen, Germany.*

*^13^Department of Dermatology, University Hospital of Tübingen, Tübingen, Germany.*

*^14^Department of General, Visceral and Transplant Surgery, University Hospital of Tübingen, Tübingen, Germany.*

*^15^Department of Clinical Pharmacology, University Hospital Tübingen, Tübingen, Germany.*

Corresponding authors: **Hans-Georg Rammensee, PhD** (rammensee@uni-tuebingen.de) and **Markus W. Löffler, MD** (markus.loeffler@uni-tuebingen.de), University of Tübingen, Interfaculty Institute for Cell Biology, Department of Immunology, Auf der Morgenstelle 15, D-72076 Tübingen, Germany

Supplementary Materials & Methods

*Dual-Luciferase Assay*

75,000 HEK 293T cells per well were transiently co-transfected in a 24-well plate with a human TLR2 plasmid (10 ng + 90 ng empty vector) or empty vector (100 ng) together with the firefly luciferase under a synthetic NF-κB promoter (100 ng) and the Renilla luciferase (100 ng) under a constitutive promoter, using Lipofectamine 2000 (ThermoFisher Scientific, Waltham, MA) according to manufacturer’s instructions. Thirty hours later, culture medium (Dulbecco’s modified Eagle medium, Invitrogen, Carlsbad, CA, with 10 % heat-inactivated FBS and 2 mM L-glutamine) was replaced and stimuli added at the stated concentrations. The cells were incubated for 18 hours and lysates were prepared and analyzed using the Dual-Luciferase reporter assay kit (Promega, Madison, MI). Pam_3_CysSK_4_ and XS15 were obtained from EMC microcollections, Tübingen, Germany.

*HEK-Dual hTLR2 analysis with TLR1, TLR2 and TLR6 blocking antibodies*

HEK-Dual hTLR2 (InvivoGen, San Diego, CA) stably express hTLR2 and produce secreted embryonic alkaline phosphatase (SEAP) driven by an NF-κB promoter. 40,000 HEK-Dual hTLR2 cells per well were seeded in a 96 well-plate and incubated for 1 h with TLR1, TLR2 and TLR6 blocking antibodies (InvivoGen) or isotype control (4 μg/ml). Then, cells were stimulated for 24 h with FSL-1 (1 ng/ml), Pam_3_CysSK_4_ (1 ng/ml), XS15 (5-10 ng/ml). Supernatants were collected and SEAP levels determined using the QUANTI-Blue detection assay (InvivoGen), as described previously (1).

*Monomers and multimers*

For monomer refolding, lyophilized peptides were diluted at 10 mg/ml in 100% DMSO immediately before use. For T-cell assays, peptides were further diluted in water/ 10% DMSO at 1mg/ml, and frozen at –80 °C. Peptides used in this study are specified in **Tables 1 & 2**. ADV-Hex HLA-A*01-peptide monomer, was generated by the conventional refolding method as described before (2,3). FLU-NCAP HLA-B*08-peptide monomer was generated by UV exchange of an HLA-B*08 UV labile monomer (containing FLRGRAJGL peptide) with ELRSRYWAI peptide, as described previously (4). Multimers were generated by incubating the monomers with streptavidin-PE or streptavidin-APC (Biolegend, San Diego, CA) at a final 4:1 molar ratio, aliquoted and frozen (-80 °C) in the presence of glycerol and human serum albumin (5).

*Dendritic cells (DC)*

Dendritic cells were differentiated from human blood monocytes. Freshly prepared PBMCs were resuspended in RPMI 1640 (Gibco, Darmstadt, Germany) supplemented with 2 % heat-inactivated human AB serum (h.i. HS, Sigma-Aldrich, Taufkirchen, Germany) and penicillin/streptomycin (Pen/Strep 100 U/ml and 0.1 mg/ml, respectively, Sigma-Aldrich) and allowed to adhere at 37 °C, 7.5 % CO_2_ (approx. 2.5 x 10^6^/condition). After 2 hours, the non-adherent cells were gently removed and adherent cells further cultured in the presence of recombinant human GM-CSF (1,000 U/ml) and interleukin-4 (25U/ml), (both PeproTech, Hamburg, Germany). After 48h, cells were either left untreated, matured with a mix of 1,000 U/ml interleukin-1β, 1,000 U/ml TNF (both PeproTech), 1 µg/ml PGE2 (Sigma-Aldrich), 200 ng/ml poly(I:C) and 1 µg/ml R848 (both InvivoGen), or treated with Pam_3_CysSK_4_ or XS15 (each 10 μg/ml, EMC).

*Immunomagnetic isolation of slanMo, NK cells, and CD4^+^ T cells*

Immunomagnetic isolation of slanMo was performed as described previously (6). Briefly, PBMCs were prepared by Ficoll-Hypaque (Biochrom) density centrifugation and incubated for 15 min at 4 °C with M-DC8 antibody containing hybridoma supernatant. After washing with PBS, 1 x 10^8^ cells were resuspended in 100 µl PBS and labeled with 15 µl of rat anti-mouse IgM coupled to paramagnetic microbeads (Miltenyi Biotec, Bergisch-Gladbach, Germany) for another 15 min at 4 °C. After washing, cells were sorted on two columns via the autoMACS device (Miltenyi Biotec). The purity of the isolated slanMo was >90% as determined by flow cytometric analysis.

CD56^+^ CD3^neg^ NK cells and CD3^+^ CD4^+^ T cells were isolated from freshly prepared PBMCs by depletion, using immunomagnetic separation according to the manufacturer´s instructions (Miltenyi Biotec). The purity of the isolated cell populations was >90% as assessed by flow cytometric analysis.

Complete medium used for culturing and functional assays consisted of RPMI 1640 (Biochrom) supplemented with 2 mM L-glutamine, 10 mM sodium pyruvate, 1 % nonessential amino acids, 100 µg/ml penicillin, 100 µg/ml streptomycin (all from Biochrom) and 10 % human serum (c.c.pro, Neustadt, Germany).

*Flow cytometric analysis*

DCs were harvested after 24 h and incubated 10 min at RT with Fc Block (BD Biosciences, Heidelberg, Germany), followed by one wash with FACS buffer (PBS with 0.02% sodium azide, 2 mM EDTA and 2% h.i. FCS) and incubation with the following mAbs for 30min at 4 °C in the dark: CD14-Alexa Fluor 700 (eBioscience, San Diego, CA), CD83-APC and CD86-BV605 (Biolegend), HLA-DR-PerCP, TLR2-PE (BD Biosciences) and Zombie Aqua (Biolegend). Cells were finally washed and fixed (FACS buffer containing 1% formaldehyde (36% w/v; Sigma-Aldrich). Fluorescence was measured on an LSR Fortessa (BD Biosciences) equipped with FACSDiva^TM^ software (Version 6.1.2). Analysis was performed with FlowJo PC version 10.

Analysis of surface molecules of slanMo, NK cells, and CD4^+^ T cells was performed using the following monoclonal antibodies. Fluorescein isothiocyanate (FITC)-conjugated anti-CD3, Phycoerythrin (PE)-conjugated anti-CD4, PE-conjugated anti-CD56, allophycocyanin (APC)-conjugated anti-human leukocyte antigen (HLA)-DR (all from BD Biosciences), and M-DC8 hybridoma supernatant (6) were used to identify immune cell subsets and to determine their purity after immunomagnetic isolation. Negative controls included directly labelled isotype-matched irrelevant antibodies (BD Biosciences). Direct immunofluorescence staining of cell surface molecules was performed using the relevant antibodies, according to the manufacturer’s instructions.

For indirect immunofluorescence staining, cells were incubated with the relevant antibodies for 15 min at 4 °C. After washing, PE-conjugated isotype-specific anti-mouse antibodies were added for 15 min at 4 °C. After the staining procedure, cells were washed twice and evaluated by flow cytometry, which was performed on a FACSCalibur flow cytometer (BD Biosciences).

For intracytoplasmic staining (ICS) of IFNγ and IL-4, CD4^+^ T cells were stimulated in the presence of 10 ng/ml phorbol myristate acetate (PMA) and 10 μg/ml ionomycin (both from Sigma-Aldrich). For blockade of exocytosis, 1 μg/ml brefeldin A was added. After 4 h, cells were harvested, fixed with freshly prepared ice-cold 4% paraformaldehyde (Merck, Darmstadt, Germany) for 15 min, and permeabilized with 0.1% saponin (Merck) in PBS containing 1 % FCS for 3 min at 4 °C. Subsequently, cells were stained for 15 min using a FITC-conjugated anti-IFNγ and a PE-conjugated anti-IL-4 antibody (both from BD Biosciences), washed twice and analyzed by flow cytometry.

*Cytokine Assay*

slanMo were plated in round-bottom 96-well plates at 5 x 10^4^/well and maintained for 6 h to allow spontaneous maturation into DCs. Then, slanMo were cultured in the presence of XS15 (10 μg/ml) for additional 18 h to stimulate cytokine secretion. In some experiments, slanMo were incubated with XS15 and IFNγ. Supernatants were collected and the concentration of TNF, IL-1β, IL-6, IL-12, and IL-23 was determined by ELISA according to the manufacturer´s instructions (BD Biosciences).

To explore whether XS15 augments the capacity of slanMo to promote IFNγ secretion by a CD8^+^ T cell clone CC7 recognizing the HLA-A*02:01-restricted WT1 peptide RMFPNAPYL (7), slanMo were maintained for 6 h to allow spontaneous maturation. Subsequently, slanMo (1 x 10^4^ cells/well) were coincubated with the CD8^+^ T cell clone (1 x 10^5^ cells/well) in the presence of the WT1 peptide and XS15 in round bottom 96-well plates. After 42 h, supernatants were collected and IFNγ was quantified.

To evaluate, whether XS15 enhances the ability of slanMo to promote IFNγ secretion by NK cells, slanMo were maintained for 6 h to allow spontaneous maturation. Subsequently, slanMo (2 x 10^4^ cells/well) were coincubated with autologous NK cells (5 x 10^4^ cells/well) with XS15 (EMC) in round bottom 96-well plates. After 42 h, supernatants were collected and IFNγ was quantified using an ELISA kit (BD Biosciences) according to the manufacturer´s instructions.

*T-cell programming*

To determine the capacity of XS15 to improve slanMo-mediated T-cell programming, slanMo were maintained for 6 h to allow spontaneous maturation. Then, slanMo (5 x 10^4^ cells/well) were cocultured with allogeneic CD4^+^ T cells (10^5^ cells/well) in the presence of XS15 (10 μg/ml). After 8 days, T cells were harvested and incubated with phorbol myristate acetate and ionomycin, which efficiently trigger intracellular cytokine expression. After 4 h, T cells were analyzed for IFNγ and IL-4 production by flow cytometry.

*Human B and NK cell activation*

Fresh PBMCs (approx. 2.5 x 10^6^/condition) were cultured in IMDM medium containing 10 % h.i. HS, Pen/Strep and 50 µM ß-mercaptoethanol either alone or with Pam_3_CysSK_4_ or XS15 (each 10 μg/ml), or a mix of phytohaemagglutinin-L (PHA) and Pokeweed mitogen (PWM) (2 and 1 µg/ml, respectively). After 40 h, adherent and non-adherent cells were harvested, washed with FACS buffer and incubated 10 min at RT with Fc Block (BD Biosciences); after one more wash, cells were stained with mAbs CD3-BV711, CD56-BV421, CD19-BV785 (all Biolegend), CD14-Alexa Fluor 700, HLA-DR-PerCP, CD69-APC-Cy7 (all BD Biosciences) and Zombie Aqua (Biolegend) for 30 min at 4 °C. Cells were finally washed twice, fixed and acquisition was performed as described above.

*Isolation of cells from the granuloma*

Forty four days after vaccination, the granuloma forming by vaccination was surgically removed along with the top layer of abdominal skin, weighing approx. 21 g. From 1/3^rd^ of this tissue, the top skin layer and fatty tissue were dislodged, leaving about 1.3 g. About 100 mg was used for *in vitro* expansion of granuloma infiltrating T cells as described below and the remaining tissue (1.2 g) was dissociated into a single cell suspension by combined mechanical and enzymatic processes. Briefly, the tissue was cut into pieces of 1–8 mm^3^ and dissociated using a mix of enzymes H and A (Tumor Dissociation Kit, Miltenyi) and a gentleMACS Dissociator (Miltenyi), following the manufacturer´s instructions. After a final filtration on a cell strainer (100 µm), cells were separated over a density gradient and 4.3 x 10^6^ cells were finally recovered. One million cells were used for phenotyping and the remaining cells were rested overnight for ELISpot assay. All the cells that were obtained from the granuloma, either by tissue dissociation or after *in vitro* expansion, are referred to as granuloma infiltrating cells (GICs).

*Phenotyping of granuloma infiltrating cells (GICs)*

After isolation, 0.5 x 10^6^ cells (GICs and PBMCs) were stained extracellularly in FACS buffer containing CD3-PE-Cy5.5 (clone SK7, eBioscience), CD4-BV711 (clone OKT4, Biolegend), CD8-PerCP (clone SK1, Biolegend), CD25-BV605 (clone BC96, Biolegend), CD45RA-BV570 (clone Hl100, Biolegend), CCR7-BV650 (clone G043H7 Biolegend), CD39-BV421 (clone A1, Biolegend), PD-1-APC-Cy7 (clone EH12.2H7, Biolegend), LAG3 (clone 17B4, Enzo Life Sciences, Lörrach, Germany), BTLA-PE (clone MIH26, Biolegend), CTLA4-PE-CF594 (clone BNI3, BD Biosciences), Tim-3-PE-Cy7 (clone F38-2E2, Biolegend) and a Live/dead-Aqua dye (Life technologies, Carlsbad, CA) or with corresponding isotype controls (except for CD3, CD4, CD8 and Live/dead-Aqua dye) derived from the same manufacturer used at the same concentration. After extracellular staining, cells were fixed and permeabilized using the Fixation/permeabilization solution (eBioscience), followed by an intracellular staining using Foxp3-FITC (clone PCH101, eBioscience) and Ki67-Alexa Fluor 700 (clone B56, BD biosciences). Cells were washed twice, fixed and the fluorescence was measured on an LSR Fortessa (BD Biosciences).

*In vitro expansion of granuloma infiltrating cells*

The granuloma tissue was cut into several pieces (~ 1 mm^3^) and one to two fragments were transferred in each well of a 24 well plate containing TIL culture medium (IMDM medium containing 7.5% h.i. human serum, 0.5% penicillin and streptomycin, 1000 U/ml human IL-2 (Proleukin, Novartis, Basel, Switzerland) and 30 ng/ml anti-CD3 antibody (clone OKT3, Miltenyi Biotec). Half of the medium was exchanged every two or three days and the cells were expanded for a total of 12 days.

*IFNγ ELISpot*

IFNγ secretion by PBMCs and GICs in response to peptide stimulation was determined using ELISpot assay, as described previously (8). Briefly, cells were re-stimulated using peptides (5 μg/ml for HLA-class I peptides and 2.5 μg/ml for HLA-class II peptides) in a 96 well ELISpot plate (MSHAN4B50, Millipore, Darmstadt, Germany), which was pre-coated with anti-IFNγ antibody (clone 1-D1K, Mabtech, Nacka Strand, Sweden). Phytohemagglutinin-L (PHA-L; 10 µg/ml) was used as a positive control and HIV-A*01, HIV-B*08 or Fil-A peptides, or DMSO 10 %, were used as negative controls (**Table 1**). 26 h later, the secreted IFN γ was detected using biotinylated anti-IFNγ antibody (clone 7-B6-1, Mabtech), Extravidin phosphatase enzyme and BCIP/NBT tablets (Sigma-Aldrich). Spots were scanned and counted with an ImmunoSpot Series 6 Core ELISpot Reader (C.T.L. Europe, Bonn, Germany). For ELISpot after *in vitro* pre-sensitization, cells were first stimulated with a pool of relevant peptides in the presence of IL-2, as previously described.

*Multimer staining*

The multimer staining protocol conformed essentially to the one suggested by CIP (http://www.cimt.eu/workgroups/cip). PBMC or GICs were centrifuged in a 96 well U bottom plate and mixed with 100 μl FACs buffer. The detailed staining procedure has been described previously (9). Multimers were diluted in multimer solution (PBS containing 0.02 % sodium azide, 2 mM EDTA and 50 % h.i. FCS) at 5 μg/ml and the cells were stained for 30 min at room temperature. Afterwards, the cells were washed and incubated for 30 min at 4 °C in 50 μl FACS buffer containing CD4-FITC (clone HP2/6, in-house production), CD8 PE-Cy7 (clone SFCI21-Thy2D3, Beckman Coulter, Brea, CA) and Aqua Live/Dead (ThermoFisher). After a 20 min final incubation step at 4 °C, cells were washed and analyzed.

*Estimation of functional vaccine specific T cells in the granuloma and in peripheral blood*

For a rough estimate of vaccine specific T cells, respective cells within the granuloma were calculated as follows:

From about 1/3 of the excised granuloma, yielding a 1.3 g piece of tissue, a 1.2 g piece was used for the isolation of granuloma infiltrating T cells (GICs), wherefrom 4.3 x 10^6^ lymphocytes could be recovered. *Ex vivo* ELISpot indicated on average 152 ADV-Hex-specific, 125 FLU-NCAP-specific and 568 EBV-GP350-specific T cells per 50,000 cells (**Fig. 4A**), that is 16,900 vaccine specific T cells per 10^6^ lymphocytes. Thus, the 1.2 g piece contained ~72,670 vaccine specific T cells, and the entire granuloma (4.9 g) approximately 300,000 vaccine specific T cells.

A blood count taken on (day 44) when the granuloma was resected indicated 1.39 x 10^6^ lymphocytes per ml of blood. Estimating a blood volume of 4,620 ml based on the volunteer´s body weight, we calculated approximately 6,400 million lymphocytes. ADV-Hex-specific IFNγ-positive T cells, tested by *ex vivo* ELISpot, were found on average at 127 spots per 300,000 cells (**Fig. 3B**), yielding 423 vaccine specific T cells per million lymphocytes (i.e. ~2.70 x 10^6^ cells for the total blood volume). For FLU-NCAP-specific IFNγ-positive T cells, respective numbers are 120 per 300,000 cells, resulting in 400 vaccine specific T cells per million lymphocytes (i.e. ~2.56 x 10^6^ cells for the total blood volume). EBV-GP350-specific IFNγ-positive T cells were on average 716 per 300,000 cells, resulting 2,387 per 10^6^ lymphocytes (i.e. ~15.27 x 10^6^ cells for the total blood volume). We therefore estimate the total number of circulating vaccine specific T cells to account for about 20.54 x 10^6^. Results from the analyzed tetramer-positive T cells produced compatible results.

*Degranulation and intracellular cytokine staining (ICS)*

ICS was performed as reported earlier (8). Briefly, cells were stimulated with individual peptides (10 μg/ml) or with an equal volume of water/ 10 % DMSO in the presence of anti-CD107a FITC (1.5 µl/ test, clone H4A3, BD Biosciences), GolgiStop (1:1500, BD Biosciences) and Brefeldin A (10 μg/ml, Sigma-Aldrich). After 12 h stimulation, cells were washed and stained with Aqua Live Dead, CD4-APC-Cy7 (BD Biosciences), CD8-PECy7 mAb (Beckman Coulter) and CD3-BV711 (Biolegend) for 20 min at 4 °C, fixed and permeabilized in Cytoperm/Cytofix (BD Biosciences) for 20 min at 4 °C and further stained with anti-IFNγ-Alexa Fluor 700 (BD Biosciences), anti-TNF-Pacific Blue (Biolegend), anti-IL-10-PE (BD Biosciences) and anti-IL-2-APC (BD Biosciences) for 30 min at 4 °C.

*Luminex multiplexed bead-based sandwich immunoassay*

Levels of 42 proteins and immune-associated markers were measured. Samples were thawed at room temperature, vortexed, spun at 18,000 g for 1 min and pipetted into a master microtiter plate for multiplexed immunoassay. The kit components of the multiplexed immunoassay were kindly provided by Myriad RBM, Austin, TX (http://rbm.myriad.com). After dilution with assay diluents in a ratio of 1:5, an aliquot of 10 µl diluted serum was introduced into one of the capture microsphere multiplexes followed by incubation at room temperature for 1 h. Reporter antibodies were added followed by incubation for an additional hour at room temperature. Streptavidin-PE solution was added for development and incubated for 1 h at room temperature. For control purposes, calibrators and controls were included on each microtiter plate. Standard curve, control and sample quality control were performed to ensure proper assay performance. Samples were tested in singles. Analysis was performed using the Luminex 100/200 instrument and data were interpreted using the software developed and provided by Myriad RBM.

*Detection of antibody responses against the vaccinated peptides and XS15*

Antibodies were detected by ELISA with an in-house assay as published previously (10). Briefly, microtiter plates were coated with XS15 and the attached peptide GDPKHPKSF. Sera from the vaccinated individual obtained at different time points were diluted 1:500 for the demonstration of IgG and IgM antibodies. Bound antibodies were detected with peroxidase conjugated goat anti-human IgG- and IgM antibodies (DIANOVA, Hamburg, Germany) at dilutions of 1:2,000. As substrate o-phenylenediamine was used. Reactivity was given as absorbance x 1,000. Optimal antigen- and serum dilutions have been evaluated by serial dilutions prior to analysis. Additionally, for controls serum from two healthy donors was tested for respective antibody responses as well as testing responses to Bacillus Calmette-Guérin (BCG) and tetanus toxoid in all samples analyzed.

*Isolation of HLA ligands from granuloma tissue and detection of vaccinated peptides by mass spectrometry*

HLA class I and HLA-DR ligands were isolated by immunoaffinity purification using the monoclonal antibodies W6/32 and L243 (both produced in-house at the Department of Immunology, University of Tübingen, Tübingen, Germany) as described previously (11). Peptides were isolated from a 0.49 g specimen from the central part of the granuloma and 20% shares of the HLA ligand extracts were analyzed by tandem mass spectrometry (LC-MS/MS) on an Orbitrap Fusion Lumos mass spectrometer online-coupled to an Ultimate3000 RSLCnano (both ThermoFisher Scientific). Peptides were separated at 50°C on a 25 cm Acclaim PepMap C18 RSLC column (ThermoFisher Scientific) using a gradient ranging from 2.4-32.0 % acetonitrile over the course of 90 min. Mass spectrometry was performed in data dependent “top speed” mode acquiring Orbitrap MS/MS spectra at 30,000 resolution for precursors of 400-650 m/z (HLA class I) and 300-900 m/z (HLA class II) with charge states 2-4+ allowed for fragmentation. Vaccinated peptides were specifically prioritized for fragmentation using a target mass list of their 2+ and 3+ precursor masses. Data processing was performed by SEQUEST database search against the reviewed Swiss-Prot human reference proteome concatenated with the vaccinated peptide sequences. Verification of IDs derived from vaccinated peptides was performed by comparing fragmentation patterns and retention times to their (isotopically labeled) synthetic counterparts (Department of Immunology), which were analyzed using 300 fmol/peptide on column and identical LC-MS settings, except that no target mass list was used.

*Transcriptome sequencing and analysis of differential gene expression*

RNASeq was performed by an external service provider (CeGaT GmbH, Tübingen, Germany). In brief, RNA was isolated from snap-frozen granuloma center, granuloma margin and distal edge normal subcutaneous tissue, using the RNeasy Mini kit (Qiagen, Hilden Germany). 100 ng of isolated RNA was employed for library preparation using the TruSeq Stranded mRNA Kit (Illumina, San Diego, CA). Single end sequencing was performed on a HiSeq 2500 instrument with a target read length of 100 base pairs and a depth of at least 50 million reads. Mapping of sequenced reads to the reference genome (hg19) was performed using STAR software (Version 2.4.0). Additional data processing and counts of mapped reads were computed with Cufflinks Tool Suite (Version 2.1.1). Resulting FPKM values were calculated using Cuffdiff employing a pooled-variance model and geometric normalization with enabled multi-read-correction (**Auxiliary Suppl. Material 1-3**). Differential expression of genes (FC>5, q<0.05) in the granuloma center vs. the margin was assessed (**Auxiliary Suppl. Material 4)** and a pre-selected gene set of interest (hallmark inflammatory response gene set, comprising 200 genes; last accessed: December 2018; http://software.broadinstitute.org/gsea/msigdb/cards/HALLMARK_INFLAMMATORY_RESPONSE.html) compared for the different regions sampled from the excised granuloma.

*Histology and immunohistochemistry*

For histological evaluation a tissue sample from the granuloma center was processed as formalin-fixed paraffin embedded tissue and blocks were cut into 3-5 µm sections and stained by hematoxylin & eosin (HE). Immunohistochemistry was performed by an automated immunostainer (BOND-MAX, Leica Biosystems, Wetzlar, Germany) according to the manufacturer´s protocols with slight modifications. Samples were stained with monoclonal antibodies recognizing CD8 (clone C8/144B; Dako, Glostrup Denmark) and CD4 (clone SP35; Cell Marque, Rocklin, CA), as well CD68 (clone PGM1; Dako) and CD20 (clone L26; Dako). Appropriate positive and negative controls were used to confirm the quality of staining. Further granulocytes were identified by typical appearance as well as mineral oil deposits (representing vaccine remnants) appearing as large vacuolar structures.

*Immunofluorescence staining of slanMo and CD8^+^ T cells*

Formalin-fixed and paraffin-embedded tissue was cut into 5 µm sections. Subsequently, these sections were deparaffinized in xylene (2 x 15 min, VWR International, Fontenay-sous-Bois, France) and hydrated by washes of graded ethanol (Berkel AHK, Ludwigshafen, Germany) to water. Tissue sections were boiled for 20 min in citrate buffer (Zytomed Systems GmbH, Berlin, Germany) at pH 6.0 for antigen retrieval. Subsequently, tissues were simultaneously stained overnight at 4°C with the monoclonal mouse anti-CD8 antibody (1:100, Dako) to evaluate infiltrating CD8^+^ T cells and the monoclonal mouse anti-slan antibody DD2 (1:10, Institute of Immunology, Medical Faculty Carl Gustav Carus, TU Dresden, Germany) to detect slanMo. For the visualization of CD8^+^ T cells, tissues were incubated with an AF633-labeled goat anti-mouse IgG antibody (ThermoFisher Scientific). slanMo were visualized by incubation with a secondary antibody solution containing goat anti-mouse IgM Biotin (1:100, Southern Biotech, Birmingham, AL), followed by the application of fluorophore AF546-labelled Streptavidin (1:500, ThermoFisher Scientific), each for 20 minutes. Then, tissues were mounted with 4,6 diamidino-2-phenylindole (DAPI)-containing AKLIDES® ANA plus medium (Medipan, Dahlewitz, Germany), coverslipped, and analyzed with a Keyence fluorescence microscope BZ-9000 (Keyence, Osaka, Japan).

**References**

1. Fuchs K, Cardona Gloria Y, Wolz OO, Herster F, Sharma L, Dillen CA*, et al.* The fungal ligand chitin directly binds TLR2 and triggers inflammation dependent on oligomer size. EMBO Rep **2018**;19(12) doi 10.15252/embr.201846065.

2. Altman JD, Moss PA, Goulder PJ, Barouch DH, McHeyzer-Williams MG, Bell JI*, et al.* Phenotypic analysis of antigen-specific T lymphocytes. Science **1996**;274(5284):94-6.

3. Chandran PA, Heidu S, Zelba H, Schmid-Horch B, Rammensee HG, Pascolo S*, et al.* A Simple and Rapid Method for Quality Control of Major Histocompatibility Complex-Peptide Monomers by Flow Cytometry. Front Immunol **2017**;8:96 doi 10.3389/fimmu.2017.00096.

4. Frosig TM, Yap J, Seremet T, Lyngaa R, Svane IM, Thor Straten P*, et al.* Design and validation of conditional ligands for HLA-B*08:01, HLA-B*15:01, HLA-B*35:01, and HLA-B*44:05. Cytometry A **2015**;87(10):967-75 doi 10.1002/cyto.a.22689.

5. Hadrup SR, Maurer D, Laske K, Frosig TM, Andersen SR, Britten CM*, et al.* Cryopreservation of MHC multimers: Recommendations for quality assurance in detection of antigen specific T cells. Cytometry A **2015**;87(1):37-48 doi 10.1002/cyto.a.22575.

6. Schäkel K, von Kietzell M, Hänsel A, Ebling A, Schulze L, Haase M*, et al.* Human 6-sulfo LacNAc-expressing dendritic cells are principal producers of early interleukin-12 and are controlled by erythrocytes. Immunity **2006**;24(6):767-77 doi 10.1016/j.immuni.2006.03.020.

7. Doubrovina ES, Doubrovin MM, Lee S, Shieh JH, Heller G, Pamer E*, et al.* In vitro stimulation with WT1 peptide-loaded Epstein-Barr virus-positive B cells elicits high frequencies of WT1 peptide-specific T cells with in vitro and in vivo tumoricidal activity. Clin Cancer Res **2004**;10(21):7207-19 doi 10.1158/1078-0432.CCR-04-1040.

8. Widenmeyer M, Griesemann H, Stevanovic S, Feyerabend S, Klein R, Attig S*, et al.* Promiscuous survivin peptide induces robust CD4+ T-cell responses in the majority of vaccinated cancer patients. Int J Cancer **2012**;131(1):140-9 doi 10.1002/ijc.26365.

9. Baumgaertner P, Jandus C, Rivals JP, Derre L, Lovgren T, Baitsch L*, et al.* Vaccination-induced functional competence of circulating human tumor-specific CD8 T-cells. Int J Cancer **2012**;130(11):2607-17 doi 10.1002/ijc.26297.

10. Glaeser L, Henes J, Kotter I, Vogel W, Kanz L, Klein R. Molecular recognition patterns of anti-topoisomerase I-antibodies in patients with systemic sclerosis before and after autologous stem cell transplantation. Clin Exp Rheumatol **2018**;36 Suppl 113(4):28-35.

11. Kowalewski DJ, Stevanovic S. Biochemical large-scale identification of MHC class I ligands. Methods Mol Biol **2013**;960:145-57 doi 10.1007/978-1-62703-218-6_12.
