# Supplementary material for: A new synthetic toll-like receptor 1/2 ligand is an efficient adjuvant for peptide vaccination in a human volunteer
Source: J Immunother Cancer. 2019 Nov 15;7:307. doi: 10.1186/s40425-019-0796-5 (PMC6858783; doi:10.1186/s40425-019-0796-5)
Supplement: Supplementary file 7 — Additional file 7: Table S1. Induction of cytokine release by XS15. Table S2. Antibody responses to XS15. [file 40425_2019_796_MOESM7_ESM.docx]

A new synthetic toll-like receptor 1/2 ligand is an efficient adjuvant for peptide vaccination in a human volunteer

Hans-Georg Rammensee ^1, 2, 3^, Karl-Heinz Wiesmüller ^4^, P. Anoop Chandran ^1^, Henning Zelba ^1^, Elisa Rusch ^1^, Cécile Gouttefangeas ^1, 2 ,3^, Daniel J. Kowalewski ^1, 5^, Moreno Di Marco ^1^, Sebastian P. Haen ^1, 2, 6^, Juliane S. Walz ^1, 2, 3, 6^, Yamel Cardona Gloria ^1^, Johanna Bödder ^1^, Jill-Marie Schertel ^7^, Antje Tunger ^7, 8^, Luise Müller ^7^, Maximilian Kießler ^7^, Rebekka Wehner ^7, 8, 9^, Marc Schmitz ^7, 8, 9^, Meike Jakobi ^10^, Nicole  Schneiderhan-Marra ^10^, Reinhild Klein ^6^, Karoline Laske ^1^, Kerstin Artzner ^1^, Linus Backert ^1,5^, Heiko Schuster ^1, 5^, Johannes Schwenck ^3, 11, 12^, Alexander N. R. Weber ^1, 3^, Bernd J. Pichler ^3, 12^, Manfred Kneilling ^3, 12, 13^, Christian la Fougère ^2, 3, 11^, Stephan Forchhammer ^13^,Gisela Metzler ^13^, Jürgen Bauer^13^, Benjamin Weide^13^, Wilfried Schippert^13^, Stefan Stevanović ^1, 2, 3^, and Markus W. Löffler ^1, 2, 3, 14, 15^

*^1^Department of Immunology, Institute for Cell Biology, University of Tübingen, Tübingen, Germany.*

*^2^German Cancer Consortium (DKTK) and German Cancer Research Center (DKFZ) partner site Tübingen, Tübingen, Germany.*

*^3^ Cluster of Excellence iFIT (EXC2180) "Image-Guided and Functionally Instructed Tumor Therapies", University of Tübingen, Germany.*

*^4^EMC microcollections GmbH, Tübingen, Germany.*

*^5^Current address: Immatics Biotechnologies GmbH, Tübingen, Germany.*

*^6^Department of Oncology, Hematology, Immunology, Rheumatology and Pulmonology, University Hospital of Tübingen, Tübingen, Germany.*

*^7^Institute of Immunology, Faculty of Medicine Carl Gustav Carus, Technische Universität Dresden, Dresden, Germany.*

*^8^National Center for Tumor Diseases (NCT), Partner Site Dresden, Germany: German Cancer Research Center (DKFZ), Heidelberg, Germany; Faculty of Medicine and University Hospital Carl Gustav Carus, Technische Universität Dresden, Dresden, Germany and Helmholtz Association/ Helmholtz-Zentrum Dresden-Rossendorf (HZDR), Dresden, Germany.*

*^9^German Cancer Consortium (DKTK), Partner Site Dresden, and German Cancer Research Center (DKFZ), Heidelberg, Germany.*

*^10^NMI Natural and Medical Sciences Institute at the University of Tübingen, Reutlingen, Germany.*

*^11^Department of Nuclear Medicine and Clinical Molecular Imaging, University Hospital of Tübingen, Tübingen, Germany.*

*^12^Werner Siemens Imaging Center, Medical Faculty, University of Tübingen, Tübingen, Germany.*

*^13^Department of Dermatology, University Hospital of Tübingen, Tübingen, Germany.*

*^14^Department of General, Visceral and Transplant Surgery, University Hospital of Tübingen, Tübingen, Germany.*

*^15^Department of Clinical Pharmacology, University Hospital Tübingen, Tübingen, Germany.*

Corresponding authors: **Hans-Georg Rammensee, PhD** (rammensee@uni-tuebingen.de) and **Markus W. Löffler, MD** (markus.loeffler@uni-tuebingen.de), University of Tübingen, Interfaculty Institute for Cell Biology, Department of Immunology, Auf der Morgenstelle 15, D-72076 Tübingen, Germany

Additional File 7:

Supplementary Tables

**The PDF file includes:**

**Table S1.** Induction of cytokine release by XS15. S3

**Table S2.** Antibody responses to XS15. S4

**Table S1. Induction of cytokine release by XS15.**

Anticoagulated whole blood from the vaccinated volunteer and two healthy donors (HD) processed in parallel, was incubated with XS15 as well as LPS and PHA/Ionomycin as positive (+ ctrl.) and medium only as negative controls (-ctrl.) and supernatant harvested after 10h. Multiplexed bead-based sandwich immunoassays were performed using a LUMINEX device with a 42-analyte panel. In case of saturation, the upper limit of quantification (ULOQ) was assigned.

|  | | | FVII | IL-1β | IL-6 | TNFα | IL-10 | IL-7 | IL-8 | MCP1 | MDC | MIP-1β |
| --- | --- | --- | --- | --- | --- | --- | --- | --- | --- | --- | --- | --- |
|  | | Unit | pg/ml | | | | | | | | | |
|  | Serum | LLOQ | 1,410 | 0.90 | 2.16 | 8.95 | 5.70 | 21.1 | 6.85 | 21.7 | 14.6 | 34 |
|  | Supernatant |  | 840 | 0.54 | 1.29 | 5.37 | 3.42 | 12.7 | 4.11 | 13.0 | 8.73 | 21 |
| Vaccinated volunteer | day 0 | neg. | 12,800 | 0.17 | n.d. | 4.03 | n.d. | n.d. | 34.60 | 71.50 | 8.66 | 266 |
|  |  | XS15 | 14,600 | 7.94 | 498.00 | 201.00 | 50.20 | 27.40 | 5,870.00 | 5,990.00 | 43.20 | 23,600 |
|  |  | Pam3Cys | 21,300 | 4.54 | 72.30 | 48.20 | 9.06 | 14.10 | 748.00 | 2,450.00 | 35.20 | 7,460 |
|  |  | LPS | 34,100 | 141.00 | 1,430.00 | 760.00 | 273.00 | 14.10 | 2,740.00 | 2,060.00 | 33.80 | >27,876 |
|  |  | pos. | 25,200 | 68.60 | >1,866.00 | 1,490.00 | 286.00 | 25.80 | 4,550.00 | 4,680.00 | 33.80 | >27,876 |
| HD 1 | | neg. | 24,500 | 0.17 | n.d. | n.d. | n.d. | n.d. | 21.00 | 67.70 | 11.70 | 91 |
|  |  | XS15 | 22,300 | 2.67 | 84.60 | 56.70 | 17.80 | 14.10 | 1,130.00 | 4,120.00 | 22.00 | 18,400 |
|  |  | Pam3Cys | 23,200 | 2.13 | 26.00 | 32.30 | 4.60 | n.d. | 360.00 | 1,430.00 | 14.50 | 4,310 |
|  |  | LPS | 32,400 | 114.00 | 776.00 | 715.00 | 108.00 | 4.67 | 1,510.00 | 1,380.00 | 28.60 | >27,876 |
|  |  | pos. | 27,700 | 64.20 | 1,110.00 | 1,130.00 | 256.00 | 14.10 | 2,270.00 | 1,570.00 | 35.70 | >27,876 |
| HD 2 | | neg. | 17,700 | 0.17 | n.d. | 4.03 | n.d. | n.d. | 35.30 | 63.60 | 19.60 | 190 |
|  |  | XS15 | 16,000 | 12.00 | 365.00 | 68.70 | 37.30 | 17.50 | 2,100.00 | 7,160.00 | 33.80 | 17,100 |
|  |  | Pam3Cys | 19,200 | 16.40 | 238.00 | 49.40 | 31.70 | 6.89 | 678.00 | 4,160.00 | 31.80 | 10,700 |
|  |  | LPS | 23,200 | 79.70 | 623.00 | 283.00 | 59.20 | 8.80 | 1,500.00 | 1,750.00 | 38.60 | 21,800 |
|  |  | pos. | 16,000 | 52.00 | 1,280.00 | 699.00 | 193.00 | 17.50 | 3,800.00 | 2,290.00 | 39.50 | >27,876 |

**Supplementary Table 2. Antibody responses to XS15.**

Antibody responses, according to different subclasses, to XS15 (GDPKHPKSF peptide as well as to the Pam3Cys moiety) of one vaccinated volunteer before the first vaccination with XS15 (pre) as well as at different time points thereafter. Additionally antibody responses of two healthy blood donors (HD1/ HD2) are shown, as well as to Bacillus Calmette-Guérin (BCG) and tetanus toxoid for comparison. Results given in bold should be considered as positive. Vx (vaccination).

|  | **GDPKHPKSF** | | | | | | **PAM_3_Cys moiety** | | | | | | **BCG** | | | | | | **Tetanus Toxoid** | | | | | |
| --- | --- | --- | --- | --- | --- | --- | --- | --- | --- | --- | --- | --- | --- | --- | --- | --- | --- | --- | --- | --- | --- | --- | --- | --- |
|  | total IgG | IgM | IgG1 | IgG2 | IgG3 | IgG4 | total IgG | IgM | IgG1 | IgG2 | IgG3 | IgG4 | total IgG | IgM | IgG1 | IgG2 | IgG3 | IgG4 | total IgG | IgM | IgG1 | IgG2 | IgG3 | IgG4 |
| **days after 1^st^ Vx** | absorbance x 1000 | | | | | | absorbance x 1000 | | | | | | absorbance x 1000 | | | | | | absorbance x 1000 | | | | | |
| pre | 80 | 40 | 176 | 128 | 78 | 22 | 150 | 126 | 448 | 396 | 58 | 20 | 240 | 92 | 62 | 380 | 306 | 32 | 86 | 48 | 536 | 494 | 36 | 130 |
| 29 | 96 | 440 | 158 | 116 | 72 | 24 | 178 | 338 | 364 | 336 | 110 | 44 | 138 | 96 | 48 | 164 | 276 | 54 | 58 | 26 | 454 | 476 | 16 | 144 |
| 44 | 114 | 524 | 204 | 162 | 96 | 40 | 220 | 432 | 696 | 438 | 134 | 60 | 140 | 88 | 54 | 158 | 250 | 40 | 82 | 58 | 580 | 550 | 28 | 142 |
| 69 | 148 | 396 | 146 | 112 | 52 | 16 | 192 | 350 | 316 | 264 | 90 | 8 | 160 | 80 | 28 | 114 | 326 | 14 | 58 | 44 | 500 | 518 | 22 | 170 |
| 119 | 260 | 282 | 234 | 136 | 36 | 44 | 158 | 236 | 416 | 326 | 92 | 46 | 134 | 74 | 100 | 144 | 248 | 36 | 68 | 42 | 510 | 502 | 54 | 162 |
| HD1 | 226 | 177 | 40 | 87 | 116 | 52 | 215 | 100 | 27 | 98 | 109 | 34 | 265 | 128 | 88 | 185 | 111 | 28 | 247 | 126 | 1094 | 1022 | 108 | 28 |
| HD2 | 475 | 39 | 145 | 157 | 44 | 9 | 200 | 61 | 32 | 79 | 53 | 12 | 213 | 35 | 51 | 69 | 45 | 0 | 322 | 31 | 1076 | 1003 | 68 | 0 |
