# Supplementary material for: A new synthetic toll-like receptor 1/2 ligand is an efficient adjuvant for peptide vaccination in a human volunteer
Source: J Immunother Cancer. 2019 Nov 15;7:307. doi: 10.1186/s40425-019-0796-5 (PMC6858783; doi:10.1186/s40425-019-0796-5)
Supplement: Supplementary file 8 — Additional file 8: Fig. S1. Impact of XS15 on slanMo-mediated T-cell polarization. Fig. S2. Impact of XS15 on slanMo-mediated IFNγ expression by WT1 peptide-specific CD8+ T cells. Fig. S3. Ex vivo phenotype of granuloma infiltrating cells (GICs). Fig. S4. Ex vivo assessment of regulatory T cells (Treg) in granuloma infiltrating cells (GICs) and PBMCs. Fig. S5. Ex vivo phenotype of checkpoint receptors in granuloma infiltrating cells (GICs) and PBMCs. Fig. S6. Detection of vaccinated peptides in granuloma tissue by mass spectrometry. [file 40425_2019_796_MOESM8_ESM.docx]

A new synthetic toll-like receptor 1/2 ligand is an efficient adjuvant for peptide vaccination in a human volunteer

Hans-Georg Rammensee ^1, 2, 3^, Karl-Heinz Wiesmüller ^4^, P. Anoop Chandran ^1^, Henning Zelba ^1^, Elisa Rusch ^1^, Cécile Gouttefangeas ^1, 2 ,3^, Daniel J. Kowalewski ^1, 5^, Moreno Di Marco ^1^, Sebastian P. Haen ^1, 2, 6^, Juliane S. Walz ^1, 2, 3, 6^, Yamel Cardona Gloria ^1^, Johanna Bödder ^1^, Jill-Marie Schertel ^7^, Antje Tunger ^7, 8^, Luise Müller ^7^, Maximilian Kießler ^7^, Rebekka Wehner ^7, 8, 9^, Marc Schmitz ^7, 8, 9^, Meike Jakobi ^10^, Nicole  Schneiderhan-Marra ^10^, Reinhild Klein ^6^, Karoline Laske ^1^, Kerstin Artzner ^1^, Linus Backert ^1,5^, Heiko Schuster ^1, 5^, Johannes Schwenck ^3, 11, 12^, Alexander N. R. Weber ^1, 3^, Bernd J. Pichler ^3, 12^, Manfred Kneilling ^3, 12, 13^, Christian la Fougère ^2, 3, 11^, Stephan Forchhammer ^13^,Gisela Metzler ^13^, Jürgen Bauer^13^, Benjamin Weide^13^, Wilfried Schippert^13^, Stefan Stevanović ^1, 2, 3^, and Markus W. Löffler ^1, 2, 3, 14, 15^

*^1^Department of Immunology, Institute for Cell Biology, University of Tübingen, Tübingen, Germany.*

*^2^German Cancer Consortium (DKTK) and German Cancer Research Center (DKFZ) partner site Tübingen, Tübingen, Germany.*

*^3^ Cluster of Excellence iFIT (EXC2180) "Image-Guided and Functionally Instructed Tumor Therapies", University of Tübingen, Germany.*

*^4^EMC microcollections GmbH, Tübingen, Germany.*

*^5^Current address: Immatics Biotechnologies GmbH, Tübingen, Germany.*

*^6^Department of Oncology, Hematology, Immunology, Rheumatology and Pulmonology, University Hospital of Tübingen, Tübingen, Germany.*

*^7^Institute of Immunology, Faculty of Medicine Carl Gustav Carus, Technische Universität Dresden, Dresden, Germany.*

*^8^National Center for Tumor Diseases (NCT), Partner Site Dresden, Germany: German Cancer Research Center (DKFZ), Heidelberg, Germany; Faculty of Medicine and University Hospital Carl Gustav Carus, Technische Universität Dresden, Dresden, Germany and Helmholtz Association/ Helmholtz-Zentrum Dresden-Rossendorf (HZDR), Dresden, Germany.*

*^9^German Cancer Consortium (DKTK), Partner Site Dresden, and German Cancer Research Center (DKFZ), Heidelberg, Germany.*

*^10^NMI Natural and Medical Sciences Institute at the University of Tübingen, Reutlingen, Germany.*

*^11^Department of Nuclear Medicine and Clinical Molecular Imaging, University Hospital of Tübingen, Tübingen, Germany.*

*^12^Werner Siemens Imaging Center, Medical Faculty, University of Tübingen, Tübingen, Germany.*

*^13^Department of Dermatology, University Hospital of Tübingen, Tübingen, Germany.*

*^14^Department of General, Visceral and Transplant Surgery, University Hospital of Tübingen, Tübingen, Germany.*

*^15^Department of Clinical Pharmacology, University Hospital Tübingen, Tübingen, Germany.*

Corresponding authors: **Hans-Georg Rammensee, PhD** (rammensee@uni-tuebingen.de) and **Markus W. Löffler, MD** (markus.loeffler@uni-tuebingen.de), University of Tübingen, Interfaculty Institute for Cell Biology, Department of Immunology, Auf der Morgenstelle 15, D-72076 Tübingen, Germany

Additional File 8:

Supplementary Figures

**The PDF file includes:**

**Fig. S1.** Impact of XS15 on slanMo-mediated T-cell polarization. S3

**Fig. S2.** Impact of XS15 on slanMo-mediated IFNγ expression by WT1 peptide-specific CD8^+^ T cells. S4

**Fig. S3.** *Ex vivo* phenotype of granuloma infiltrating cells (GICs). S5

**Fig. S4.** *Ex vivo* assessment of regulatory T cells (T_reg_) in granuloma infiltrating cells (GICs) and PBMCs. S6

**Fig. S5.** *Ex vivo* phenotype of checkpoint receptors in granuloma infiltrating cells (GICs) and PBMCs. S7

**Fig. S6.** Detection of vaccinated peptides in granuloma tissue by mass spectrometry. S8

**
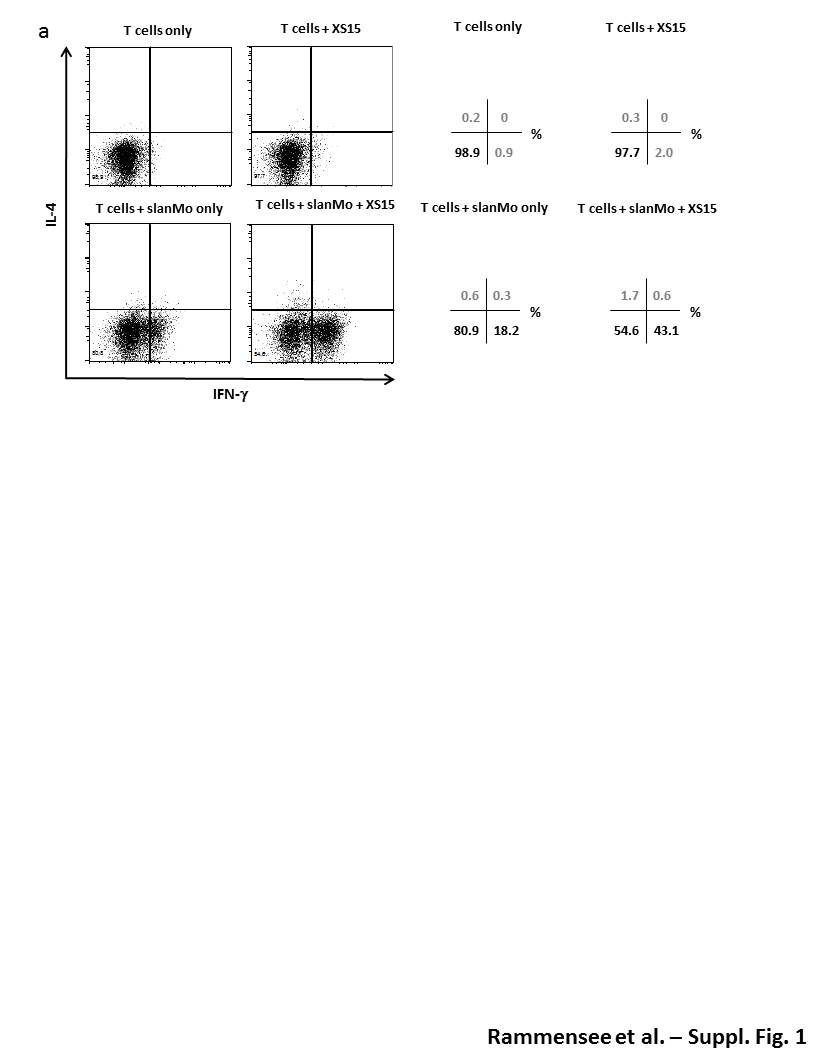
**

**Fig. S1.** **Impact of XS15 on slanMo-mediated T-cell polarization.**

slanMo were maintained for 6 h to allow spontaneous maturation. Subsequently, slanMo were coincubated with allogeneic CD4^+^ T cells in the presence of XS15. After 8 days, the percentage of IFNγ- and IL-4-producing CD4^+^ T cells was determined by flow cytometry. The results of one representative donor out of three showing similar results are depicted.

**Figure S2. Impact of XS15 on slanMo-mediated IFNγ expression by WT1 peptide-specific CD8^+^ T cells.** Immunomagnetically isolated slanMo from PBMCs of two healthy donors were maintained for 6 h to allow spontaneous maturation. Subsequently, slanMo were coincubated with the WT1 peptide-specific CD8^+^ T cell clone CC7 in the presence of the WT1 peptide and XS15. After 14 h, PMA (10ng/ml), Ionomycin (1µg/ml), and Brefeldin (1µg/ml) were added for additional 4 h. Subsequently, the percentage of IFNγ-producing slanMo and CD8^+^ T cells was determined by flow cytometry.


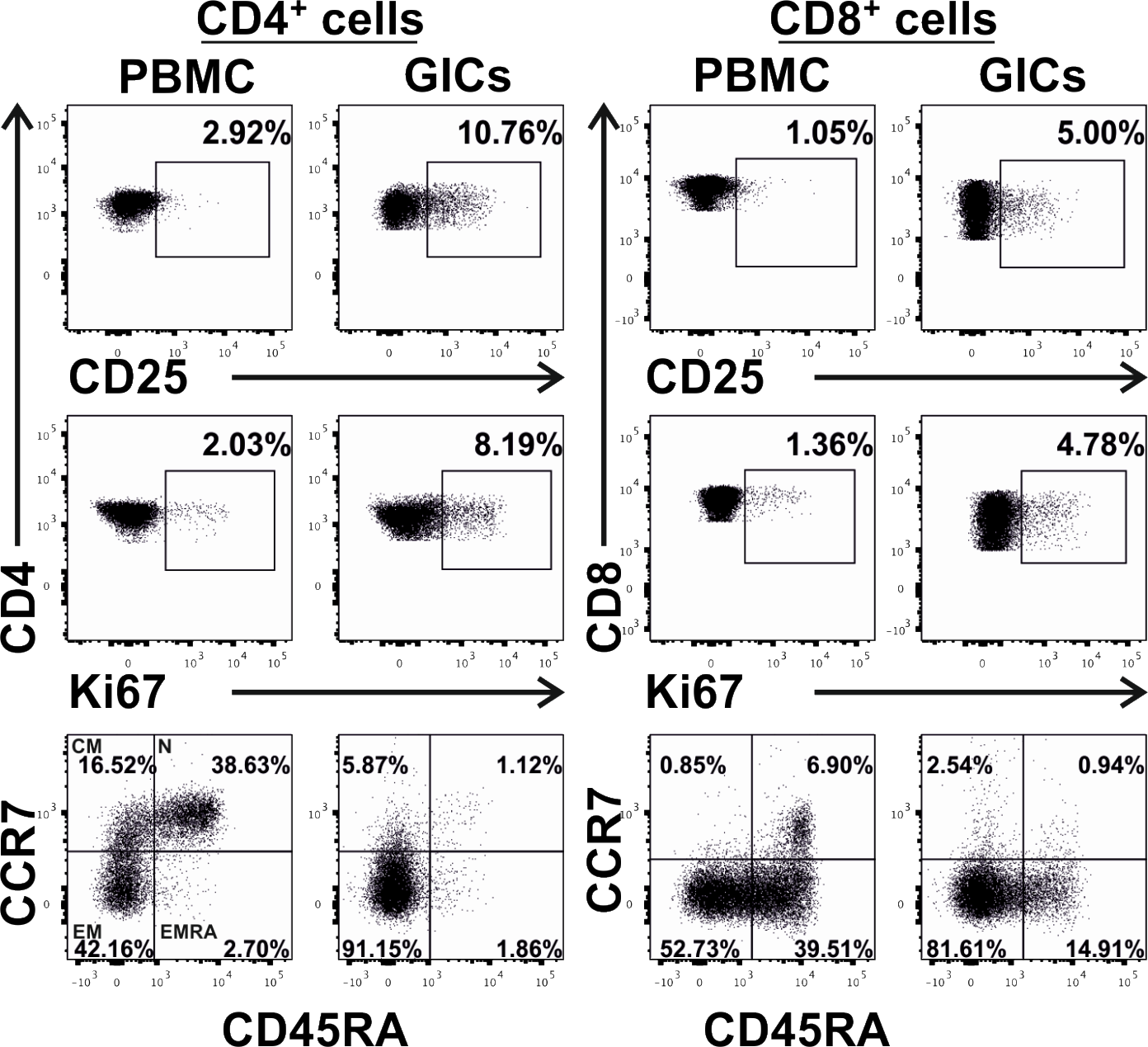


**Fig. S3.** ***Ex vivo* phenotype of granuloma infiltrating cells (GICs).** The *left panel* shows CD4^+^ cells and the *right panel* shows CD8^+^ cells within PBMCs and GICs. The *top row* shows the frequency of CD25^+^ cells and the *middle row* shows the frequency of Ki67^+^ cells. The *bottom row* shows the differentiation status of CD4^+^ non-T_regs_ (*left panel*), and CD8^+^ cells (*right panel*). N- naive (CD45RA^+^ CCR7^+^), CM- central memory (CD45RA^-^ CCR7^+^), EM- effector memory (CD45RA^-^ CCR7^-^) and EMRA- terminally differentiated effector memory RA^+^ cells (CD45RA^+^ CCR7^-^) in flow cytometry analyses; % are indicated.

**
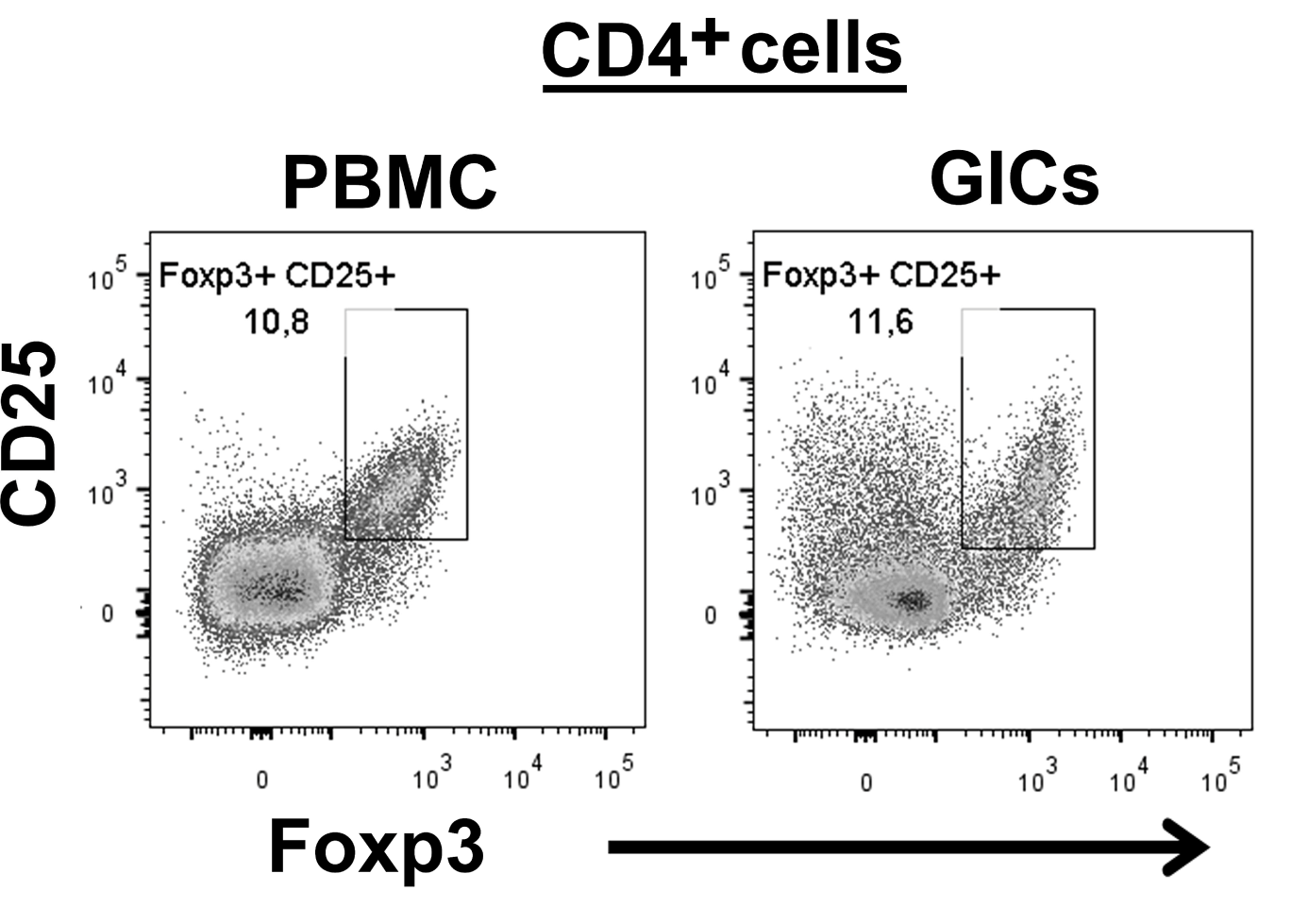
**

**Fig. S4. *Ex vivo* assessment of regulatory T cells (T_reg_) in granuloma infiltrating cells (GICs) and PBMCs.** The *panel* shows the percentage of CD25^+^ Foxp3^+^ cells among CD3^+^ CD4^+^  lymphocytes assessed in PBMCs on the *left* and respective findings for GICs on the *right* analyzed by flow cytometry; % of gated cells are indicated.

**
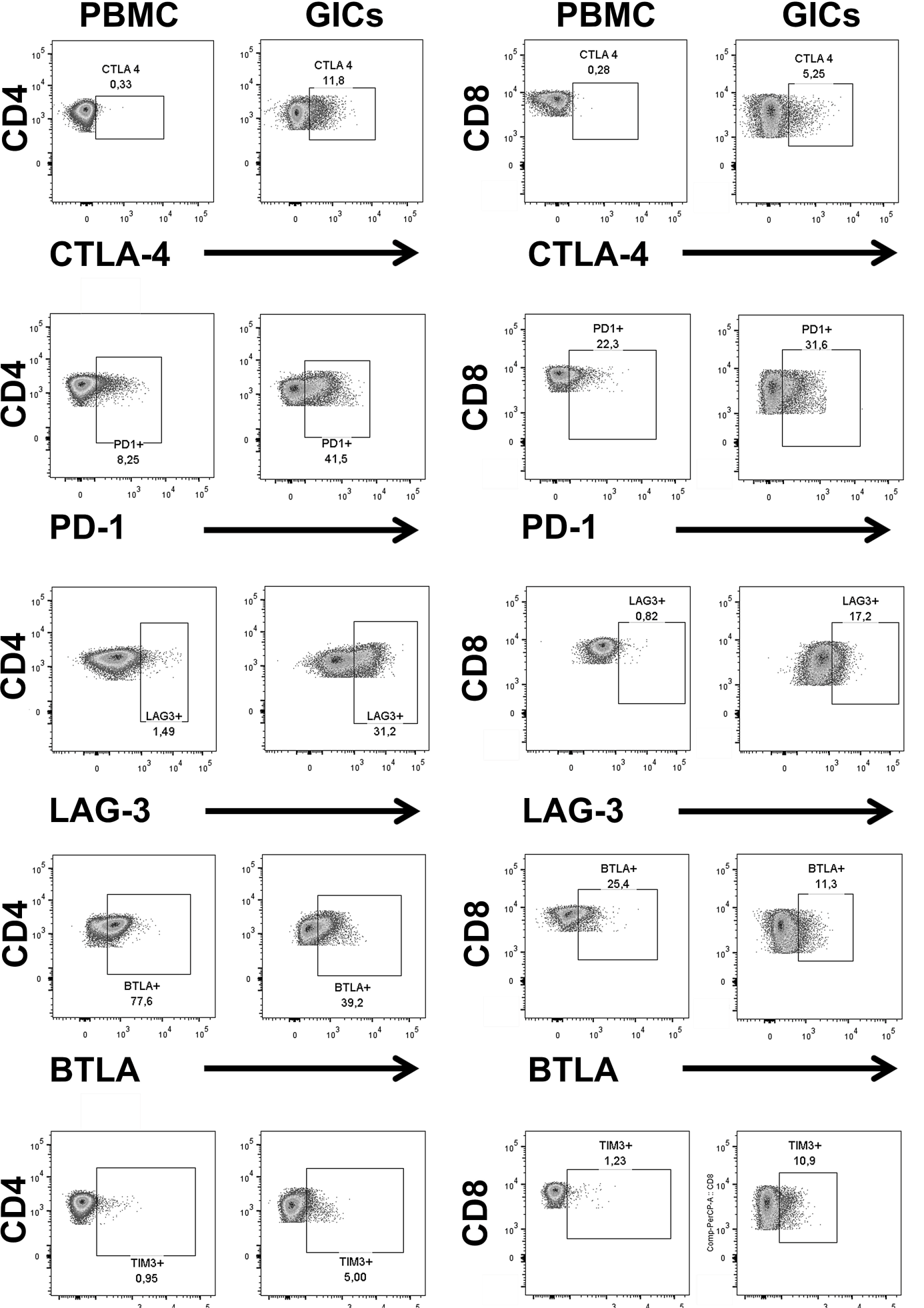
**

**Fig. S5.** ***Ex vivo* phenotype of checkpoint receptors in granuloma infiltrating cells (GICs) and PBMCs.**The *left panel* shows non T_reg_ CD4^+^ cells and the *right panel* shows CD8^+^ cells within CD3^+^ lymphocytes assessed in PBMCs and GICs respectively. Frequencies of CTLA-4^+^/ PD-1^+^/ LAG-3^+^/ BTLA^+^ and TIM-3^+^ cells are shown in flow cytometry analyses; % of gated positive cells are indicated. Gates for the checkpoint receptors were placed according to isotype controls antibodies.

**
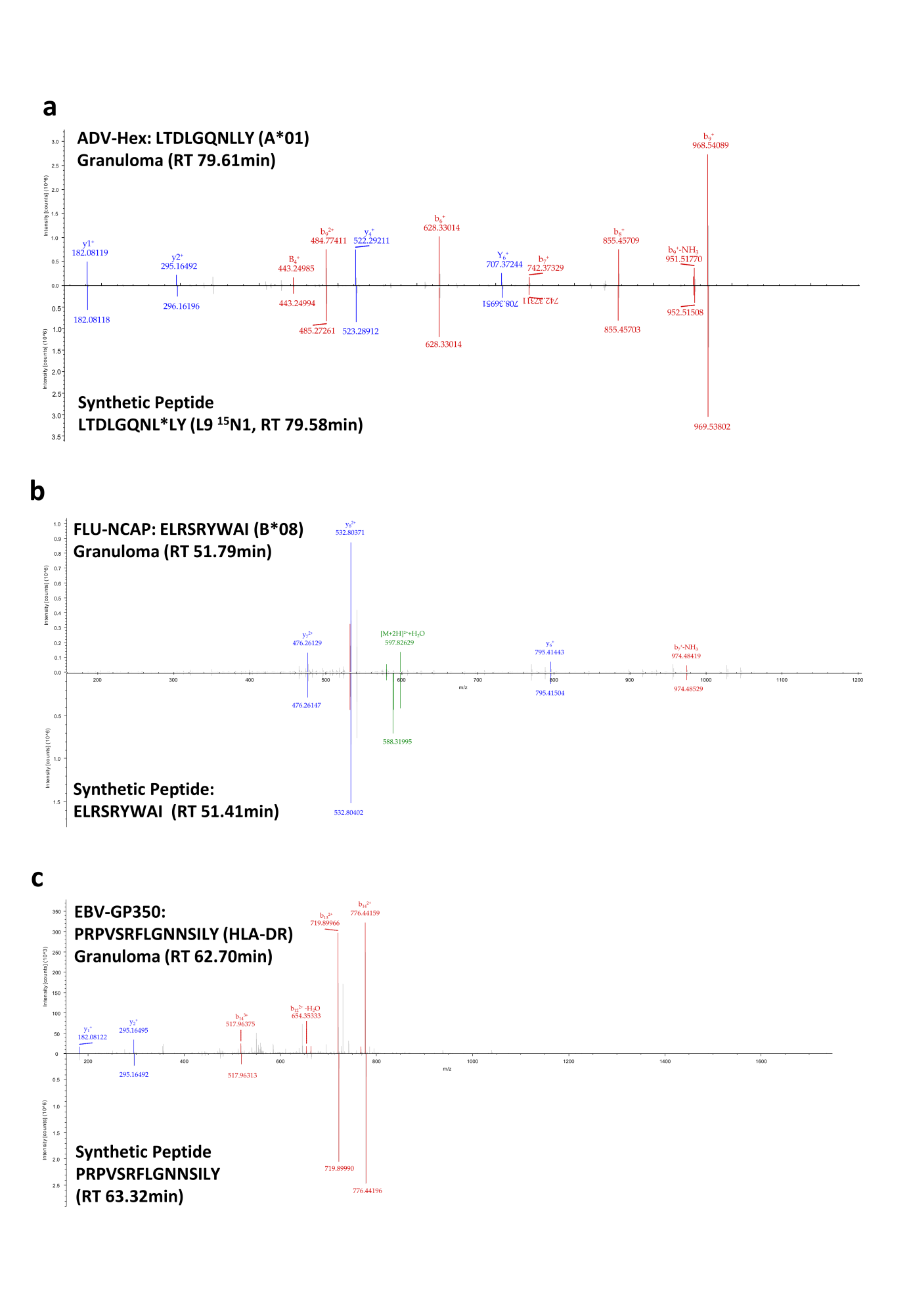
**

**Fig. S6**. **Detection of vaccinated peptides in granuloma tissue by mass spectrometry.** HLA class I and II ligands were isolated from tissue sample from the granuloma core (0.49g), using the class I-specific mAb W6/32 as well as DR-specific L243 and analysed by LC-MS/MS. Database search identified peptide-spectrum matches for all three vaccinated peptides (upwards axis), which were verified by comparison to their synthetic counterparts (downwards axis). a) ADV-Hex, (LTDLGQNLLY); b) FLU-NCAP, (ELRSRYWAI); c) EBV-GP350, (PRPVSRFLGNNSILY).
